# Ab-initio heat transport in defect-laden quasi-1D systems from a symmetry-adapted perspective

**DOI:** 10.1038/s41524-025-01866-1

**Published:** 2026-01-09

**Authors:** Yu-Jie Cen, Sandro Wieser, Georg K. H. Madsen, Jesús Carrete

**Affiliations:** 1 Institute of Materials Chemistry, TU Wien, Vienna, Austria; 2 Instituto de Nanociencia y Materiales de Aragón, CSIC-Universidad de Zaragoza, Zaragoza, Spain

**Keywords:** Materials science, Nanoscience and technology, Physics

## Abstract

Nanotubes, with their high aspect ratio and tunable thermal conductivities, are promising nanoscale heat-management components. However, their performance is often constrained by thermal resistance arising from structural defects or interfaces. Here, we examine how structural symmetry influences thermal transport through defect-laden sections. We introduce a framework that integrates representation theory with a mode-resolved Green’s function approach, enabling symmetry-resolved analysis of phonon transmission in quasi-1D systems. To capture the intrinsic symmetries of such systems and avoid artifacts, we employ line-group theory, which introduces quantum numbers that partition phonon branches into symmetry-defined subsets for clearer mode classification. Force constants and phonons are obtained using an Allegro-based machine-learning potential with near-ab initio accuracy. Applying this to single- and multi-wall MoS_2_-WS_2_ nanotubes, we link transmission probabilities of individual modes to structural symmetry. Counterintuitively, strong symmetry breaking can enhance heat transport by relaxing selection rules and opening additional transmission channels. Molecular dynamics confirms that this behavior persists even when anharmonicity is considered. The fact that higher disorder introduced through defects can enhance thermal transport, and not just suppress it, demonstrates the critical role of symmetry in deciphering the nuances of nanoscale thermal transport.

## Introduction

Transition metal dichalcogenide (TMD) nanotubes have been extensively studied due to their unique physical properties. Their widely tunable band gap makes them highly suitable for optoelectronic devices, field-effect transistors^1,2^, and water-splitting photocatalysts^3,4^. In recent years, with the development of experimental techniques, heterostructures of TMD nanotubes have been synthesized successfully^5^, showing that multi-layer TMD nanotubes can exist as stable systems.

Quasi-1D materials are natural candidates for applications in heat dissipation nanodevices^6,7^, for thermoelectric energy conversion^8^, or in various other electronic devices^9^. Many relevant applications involve heat transfer, which makes it highly relevant to study the origins of the underlying transport processes.

Many nanotube systems can reach very high thermal conductivity values, sometimes even exceeding the values observed in their 2D monolayer counterparts, as has been shown for WS_2_ nanotubes^10^ or, more extensively, for carbon nanotubes^11^. Conversely, structural modifications such as specific chiralities, the length and thickness of the wire, and the introduction of defects, interfaces, or other imperfections can drastically limit thermal transport channels. Experiment alone often faces confounding factors like different nanotube orientations or packing conditions, making it challenging to pinpoint the origin of differences in the observed properties^11^. Computational studies can help by enabling the study of those important heat transport roadblocks in much more detail. Most simulations have tackled the influence of defects in the context of molecular dynamics (MD)^10,12,13^, which is usually costly to conduct at scale and often not easy to connect to the physics of thermal carriers. More comprehensive insights can be gained at a cheaper computational cost by specifically analyzing lattice-dynamical properties^14–16^, which will be our primary focus in the following.

The calculation of thermal resistances across defect-laden regions is an area with a long development history, starting with simple continuum approximations for interfaces (the acoustic and diffuse mismatch models) and progressing towards predictive strategies based on an atomistic picture in the context of molecular or lattice dynamics^17^. Among the latter, the atomistic Green’s function (AGF) method is a powerful tool to study elastic phonon scattering by such breakdowns of periodicity. The first practical implementations of the methods targeted only the total energy transmission coefficient across defects^18^. More recently, using the concept of the Bloch matrix, Ong et al. developed the mode-resolved phonon transmission that can discriminate between the contributions of individual phonon modes^19,20^. An extension of the method has also been proposed that incorporates anharmonicity into the AGF by adding a many-body self-energy term in the calculations^21^.

Quasi-1D systems pose specific difficulties for the applications of the AGF method. Common lattice-dynamics workflows, designed and developed for 3D systems, only take advantage of translational symmetry to classify the vibrational modes; thus, when applied to a nanotube, they yield a very large number of uncategorized phonon bands^22^. Likewise, they only partially account for the continuous symmetries of free space and therefore introduce significant artifacts into those bands, rendering the results ill-suited for subsequent calculations^22,23^. Finally, those workflows typically involve symmetry detectors developed for fully periodic systems, which cannot identify rotational orders other than 2, 3, 4, and 6. All of these issues can be avoided by basing the study of vibrations in quasi-1D systems on line groups^24^, i.e., the products of a single generalized translation and a point group, rather than the more usual space groups. In particular, group projection techniques^25–27^ can provide new quantum numbers that split the set of phonon branches into much smaller subsets, each of which transforms according to a well defined irreducible representation (or irrep) of the line group, avoiding cross-contamination artifacts. This classification, based on fundamental physical features that would otherwise stay hidden, can be carried over to other quantities derived from those vibrational properties.

For nanotubes, and especially multi-layer nanotubes, the large number of atoms in the structure is another limitation that restricts the use of workflows based on first principles. Machine-learning interatomic potentials (MLIPs) provide a powerful tool for overcoming those limitations^28^. Some of the cutting-edge MLIPs in terms of economy of training data and transferability are MACE^29^, NequIP^30^ and Allegro^31^, all using equivariant neural networks. The lowered cost of evaluating energies and forces using those potentials makes it possible to obtain relatively long MD trajectories using the same dynamical model as in the AGF method, and thus assess the effects of anharmonicity and temperature on the thermal transport coefficients.

In this paper, we train a machine-learning potential for multi-layer MoS_2_-WS_2_ nanotubes and use it to obtain the interatomic force constants (IFCs), i.e., the mass-reduced second- and higher-order derivatives of the potential energy with respect to atomic displacements, of their pristine and defect-laden configurations. We then use the mode-resolved AGF method to calculate the phonon transmission across those defects and the associated thermal conductance. We enforce all continuum and line-group symmetries on the IFCs to remove any artifacts stemming from their violation, and employ symmetry-adapted bases in our calculations to keep track of which irreps are involved in scattering processes and relate those observations to symmetry breakdowns. We show that less symmetric defects, even though intuitively introducing more disorder, can in fact strengthen thermal transport by opening up previously forbidden channels for phonons. We then carry out finite-temperature MD calculations to check that the systems retain their symmetry and that anharmonicity does not change our qualitative conclusions about the correlation between symmetry and thermal transport.

## Results

In this section, we firstly demonstrate the reliability of the MLIP by evaluating the error ranges in its energy and force predictions. Next, we illustrate the significance of irreps and quantum numbers in phonon vibrational modes using a pristine single-walled nanotube as an example, and then we calculate the symmetry-adapted transmission of a pristine double-walled nanotube. Finally, we investigate defect-laden double-walled nanotubes and show that the breakdown of symmetry can actually open up more transmission channels and enhance the thermal conductance. We analyze this situation in detail through our symmetry-adapted AGF approach.

### Energy and force errors from our MLIP

**Fig. 1:**
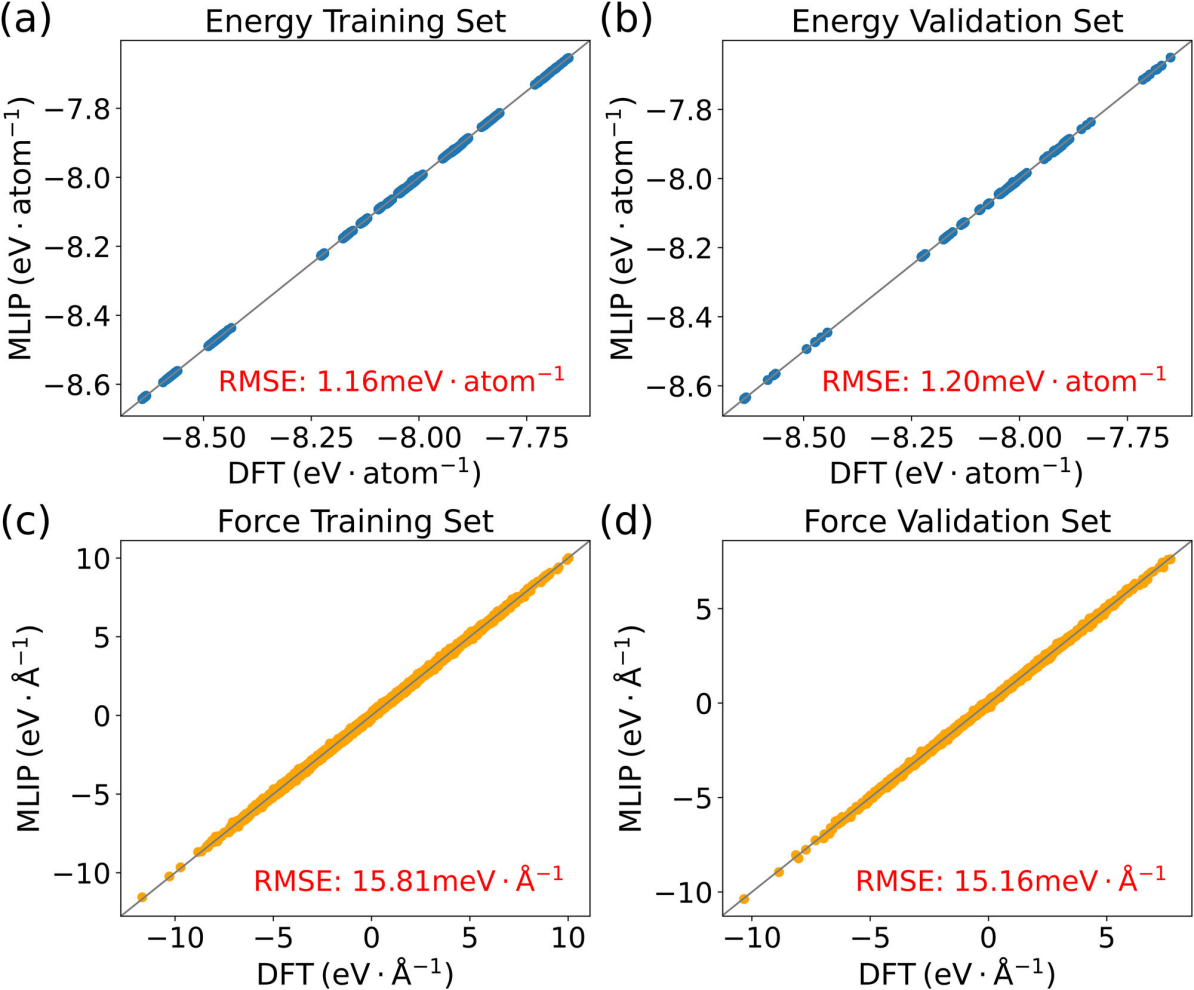
Parity plots to illustrate the accuracy of the MLIP results compared to the DFT ground truth. The comparison for the energies is given in (**a**) for the training set and in (**b**) for the validation set. Analogous comparisons for the forces are shown in (**c**, **d**). The respective root-mean-square errors are given in red text.

As shown in Fig. 1, the high accuracy of the MLIP is reflected in root-mean-square errors (RMSE) of 1.16 meV atom^−1^ and 15.81 meV Å^−1^ for the training energies and training forces, respectively. Over the validation set, the RMSEs for energies and forces are 1.20 meV atom^−1^ and 15.16 meV Å^−1^, which does not suggest any overfitting. Figure 1 shows a more detailed view, in the form of parity plots for the energies and forces over both sets. Representative errors in the forces are well below, for instance, typical discrepancies between different DFT implementations^32^. Thus, those forces are suitable for calculating the IFCs, taking into account the symmetry corrections outlined in the subsection about interatomic force constants of the Methods section.

Since we use the same MLIP for our MD simulations, it is also important to assess its precision and accuracy in that setting. To do so, we sampled 50 states from room-temperature trajectories of representative pristine and defect-laden systems, and calculated the energies and forces for those configurations using both the MLIP and direct DFT calculations. The agreement is still sufficient to evaluate the quality of the MLIP as DFT-like, with only a slight degradation in the prediction of the energies for the defect-laden case, in the form of a mostly configuration-independent offset that may originate in the DFT calculations themselves. More detailed results are presented in the supplementary information.

### Vibrational modes of a pristine single-walled WS_2_ nanotube

The irrep that each mode belongs to reflects how it changes under each of the symmetry operations, and therefore identifies the nature of the atomic displacements in that mode. Besides the linear quasimomentum *q*, expressing how the phase of the eigenvector changes under linear translations along the nanotube axis, each irrep is also characterized by an integer value of *m* describing its behavior under rotation. Irreps are further subdivided according to parities *Π*_V_.

**Fig. 2:**
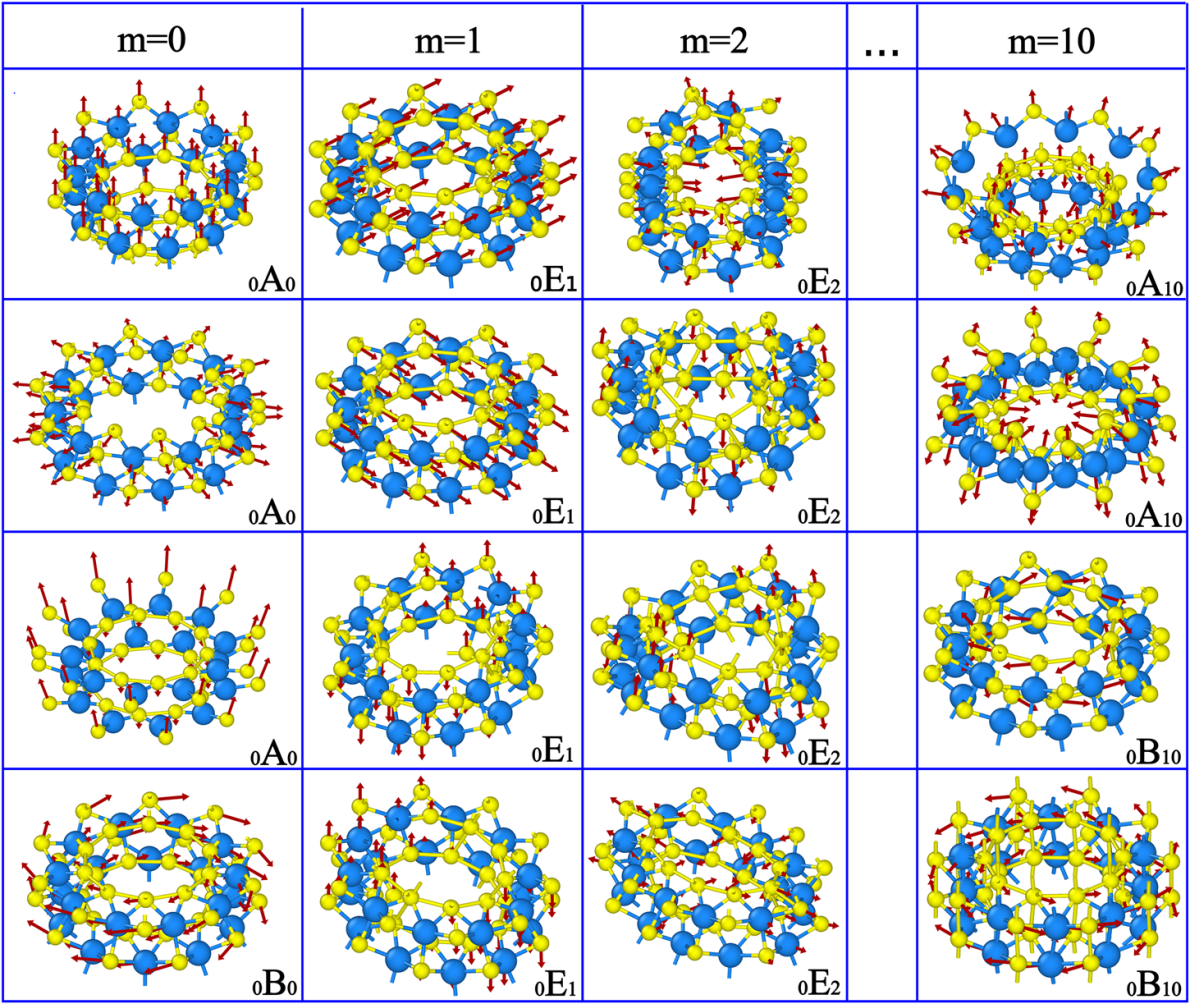
Phonon modes of a pristine single-walled WS_2_ nanotube at the *Γ* point. The modes are associated to four different values of *m* at *q* = 0. The atomic visualizations were created with the OVITO software package^62^.

To illustrate this more concretely, we consider the phonon modes of a WS_2_ nanotube with chirality (10,0), which belongs to the *L*2*n*_10_*mc* group, from the eighth of the thirteen line group families^24^. Focusing on the *Γ* point (*q* = 0), we plot a selection of vibrational modes with different *m* in Fig. 2. There are three types of irreps at that point: _0_A_0/10_, _0_B_0/10_, and _0_E_*m*_ (with 1 ≤ *m* ≤ 9). Both _0_A_0/10_ and _0_B_0/10_ are non-degenerate one-dimensional irreps; A-type modes have even parity for reflections with respect to a vertical plane (i.e., one containing the *OZ* axis), whereas B-type modes have odd parity. The first three vibrational modes for *m* = 0 and the first two for *m* = 10 correspond to A-type irreps, combining axial expansion and contraction (breathing) with longitudinal vibrations; the last mode for *m* = 0 and the last two for *m* = 10 belong to B-type irreps and correspond to shear and torsion modes. On the other hand, _0_E_*m*_ is a doubly degenerate two-dimensional irrep that arises in systems with *C*_*n*_ rotational symmetry. The degeneracy reflects vibrations along two possible orthogonal directions. All vibrational modes for 1 ≤ *m* ≤ 9 are bidirectional stretching modes belonging to E-type irreps.

### Spectrum and transmission of a pristine double-walled WS_2_-MoS_2_ nanotube

The translational block of our model double-walled WS_2_-MoS_2_ nanotube contains 40 Mo, 20 W, and 120 S atoms and has chirality (10, 0) − (20, 0). It belongs to the *Lnmm* line group, part of the sixth line-group family. There is no interface scattering, so the transmission coefficient at a given frequency equals the number of available phonon modes. By employing our symmetry-adapted AGF approach, the phonons can be categorized into different irreps, allowing a detailed analysis of each irrep’s contribution to the transmission coefficient.

**Fig. 3:**
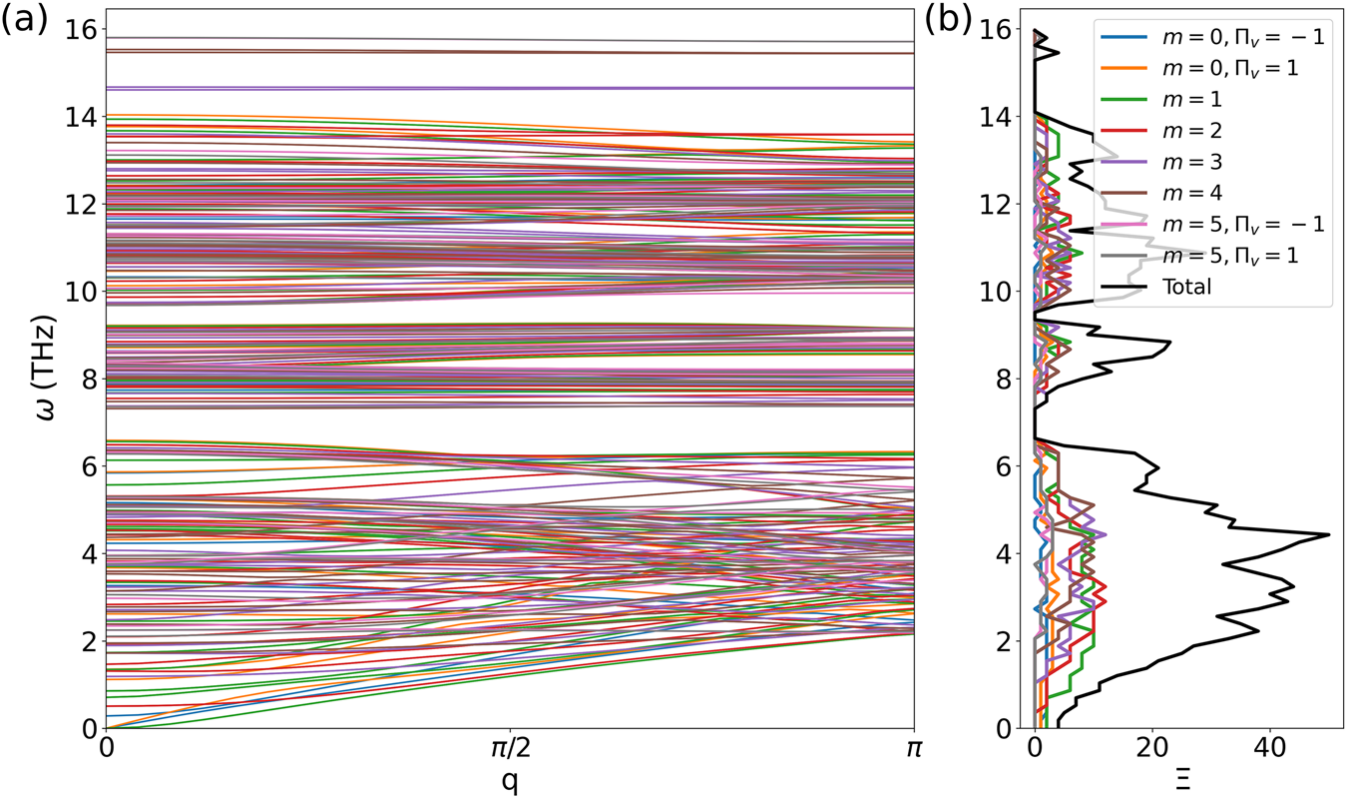
Symmetry-adapted phonon dispersions and transmission for a pristine double-walled WS_2_-MoS_2_ nanotube. The phonon dispersions as a function of wave number are shown in (**a**), and the transmission in (**b**). Different colors correspond to different irreps, classified by angular quasimomentum *m* and parity *Π*_V_. The black line indicates the total transmission.

The results are presented in Fig. 3. There are two degenerate quadratic ZA branches, which belong to *m* = 1 and irrep _0_E_1_. The two remaining acoustic branches are linear and correspond to *m* = 0. The irrep corresponding to the LA branch (with a larger sound velocity) is _0_A_0_ and the irrep corresponding to the TA branch (with a smaller sound velocity) is _0_B_0_. The presence of four acoustic branches, two of which are quadratic, indicates that the symmetrization of the force constants was performed correctly, since otherwise only three acoustic branches would have emerged from the bulk-like workflow^22^.

Analogously to how, by breaking translational symmetry in a bulk system, a defect can cause scattering between phonon modes with different wave vectors, the breakdown of the symmetries making up the line group can cause scattering between irreps, potentially involving not only *q* but also *m* and the parities if applicable. We explore this issue in the next subsection.

### Defect-laden double-walled WS_2_-MoS_2_ nanotube

**Fig. 4:**
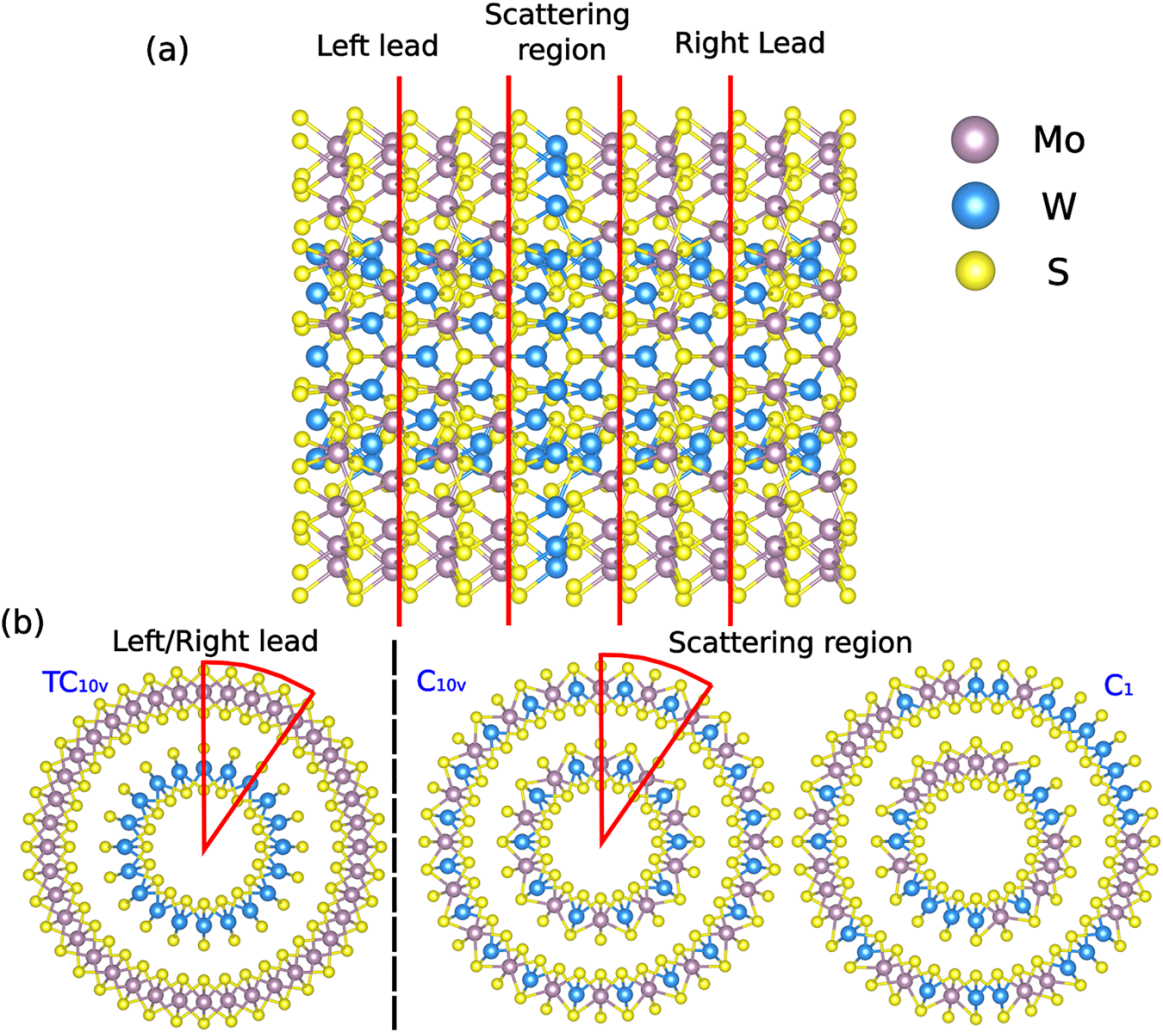
Depiction of the double-walled nanotube systems including the defect-laden scattering region. **a** Longitudinal view of all segments used in the AGF scattering calculations. **b** Cross-sectional views of the pristine leads and of the two defect-laden configurations with different symmetries. The atomic visualizations were created with the VESTA software package^63^.

For a double-layer WS_2_-MoS_2_ nanotube with a combination of chiralities (10,0)-(20,0), the line group is *TC*_10*v*_. In this application of the symmetry-adapted AGF method, both the left and right leads are composed of the same pristine structure (40 Mo, 20 W, and 120 S atoms in each slice), while the scattering region contains the defects. To investigate how symmetry influences heat transfer, we compare two defect-laden configurations with different symmetries in the scattering region. As shown in Fig. 4, we replace 20 Mo atoms with 20 W atoms in the outer-layer nanotube and 10 W atoms with 10 Mo atoms in the inner-layer nanotube. We specifically identify sets of equivalent atoms so as to create a configuration that preserves the *C*_10*v*_ symmetry (bottom center). Meanwhile, we generate a completely asymmetric *C*_1_ configuration (bottom right) by random replacement. To ensure the comparison is affected only by symmetry, we choose exactly the same number of substitutional atoms in each nanotube in both cases. Access links to the corresponding structures are provided in the “Data availability” statement.

The second-order IFC matrices for these two defect configurations are computed employing the MLIP and used as inputs to the AGF. The total transmission coefficient *Ξ*(*ω*) and the thermal conductances *σ*(*T*) are calculated using the Caroli formula and then integrating the transmission over frequencies—see the “Methods” section for details.

**Fig. 5:**
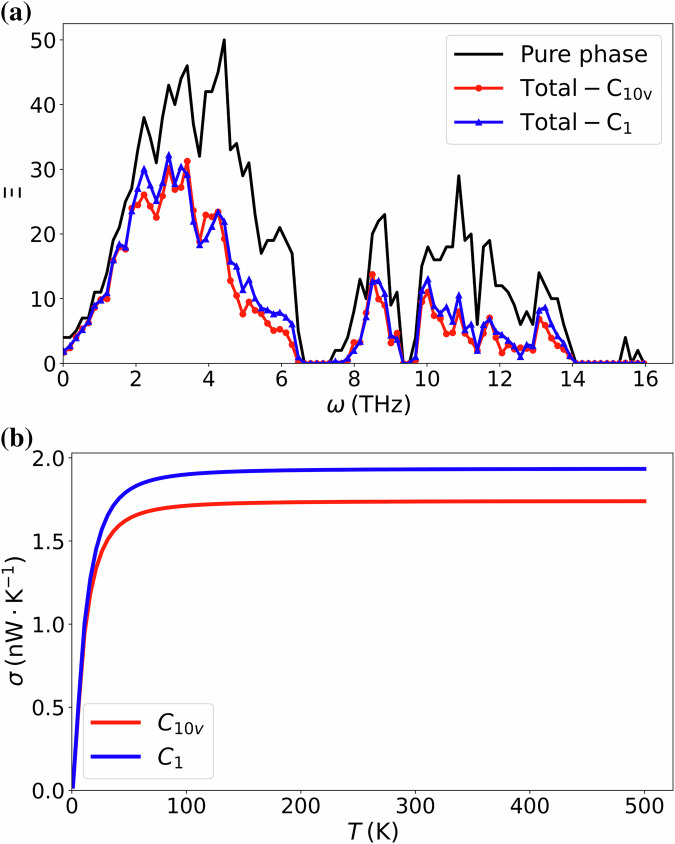
Transmission of (10,0)-(20,0) WS_2_-MoS_2_ double-walled nanotubes with a defect-laden segment with either *C*_10*v*_ or *C*_1_ symmetries. **a** Transmission coefficient spectra as a function of frequency for the pristine system (black), the structure with the symmetric defect (red), and the structure with the asymmetric defect (blue). **b** Temperature dependence of the thermal conductances for the two defect-laden structures.

As shown in Fig. 5a, we find that the transmission coefficient of the *C*_10*v*_ structure is lower than that of the *C*_1_ structure in most of the frequency range. The thermal conductance for these two configurations is presented in Fig. 5b; the results align with the transmission coefficients, with the thermal conductance of the *C*_1_ configuration also higher than that of the *C*_10*v*_ structure. This result is somewhat counterintuitive, as it is generally expected that increased disorder at the interface will lead to a stronger scattering effect and, consequently, lower thermal conductance. The mode-resolved information is key to analyzing this surprising result.

**Fig. 6:**
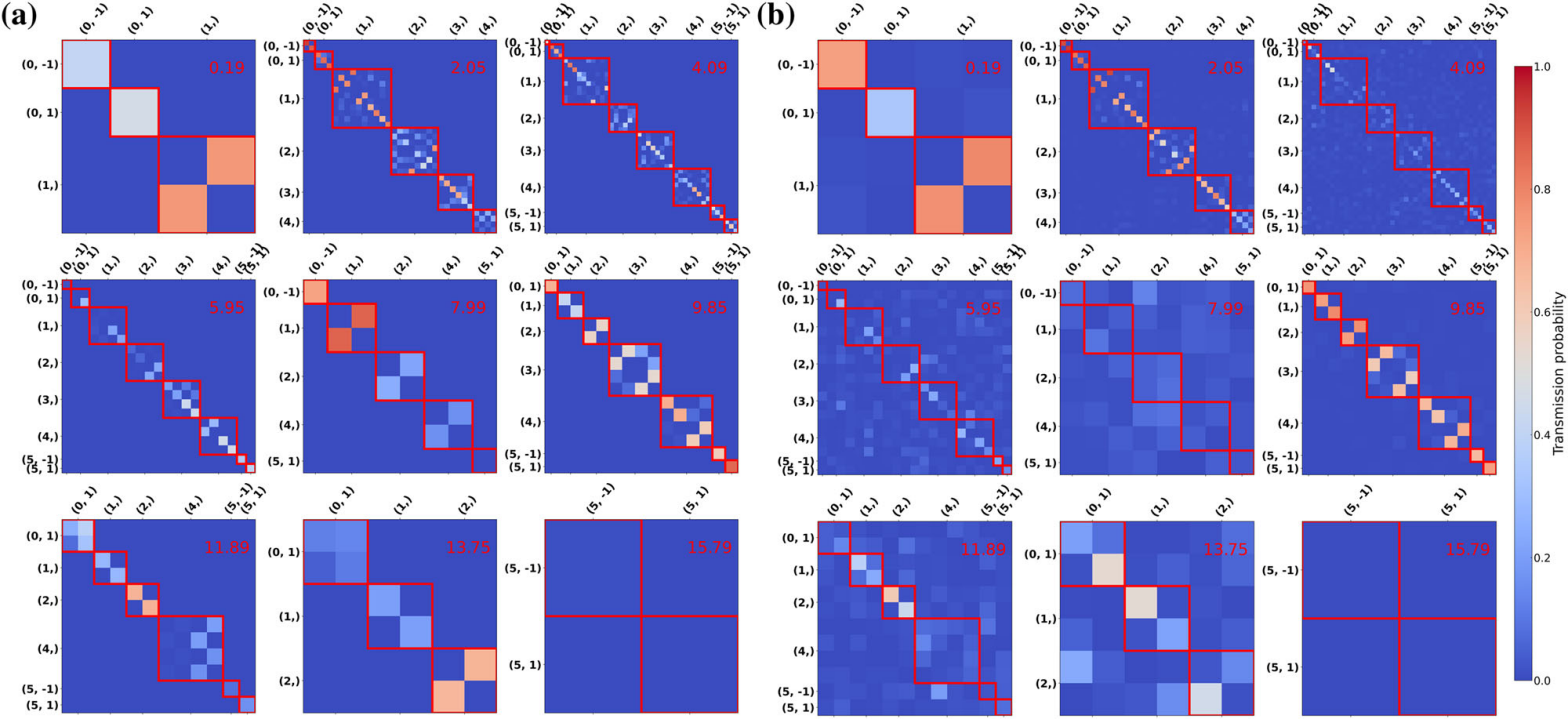
Mode-to-mode phonon transmission matrices of the defect-laden nanotubes. **a** Mode-to-mode transmission matrix of the structure containing a *C*_10*v*_ defect-laden segment. **b** Transmission matrix of the structure containing a *C*_1_ defect-laden segment. The red number in the upper-right corner of each matrix corresponds to the angular frequency of the incident and scattered phonons, in rad ps^−1^. The element *t*_*ij*_ in the transmission matrix represents the transmission probability from phonon mode *i* in the left lead to the mode *j* in the right lead. The pair (*m*, *Π*_V_) on the axes indicates the irrep, and the red square outlines groups of matrix elements corresponding to incident and transmitted modes within the same irrep.

After obtaining symmetry-adapted sets of vibrational eigenmodes for both leads, we compute a detailed transmission matrix between them following the procedure explained in the Methods section. The results are shown in Fig. 6, where each element *i*, *j* represents the transmission probability $| {{\boldsymbol{t}}}_{{\rm{LR}}}{| }_{ij}^{2}$ from phonon mode *i* in the left lead to phonon mode *j* in the right lead. The diagonal blocks (outlined with red squares) correspond to incident and transmitted modes belonging to the same irrep, and rows and column labels specify which irrep that is. If the interface (scattering region) does not break the symmetry, the harmonic matrix of that central region, ***H***_*C*_, can be block-diagonalized based on the irreps. This implies that there is no interaction between different irreps, and the heat cannot be transferred between channels from different irreps. However, when the symmetry is broken, this restriction does not apply anymore. This behavior is evident in Fig. 6: the non-zero transmission values are confined within the red squares for the *C*_10*v*_ interface [Fig. 6a]. In contrast, for the *C*_1_ interface [Fig. 6b], some transmission appears outside the red square.

The most salient of the symmetry-induced selection rules evidenced by Fig. 6 through their fulfillment and breakdown is the conservation of *m*, or of the complete angular quasimomentum. While the *C*_10*v*_-symmetric defect arrangement respects the rotational part of the line group and therefore does not mix irreps with different *m*, the *C*_1_ configuration completely destroys the rotational symmetry and is thus incompatible with normal modes with a well-defined *m*. This is apparent, for instance, in the transmission matrix for phonons of frequency 5.95 rad ps^−1^ in the less symmetric defect-laden configuration, with numerous visibly non-zero elements connecting blocks with different values of *m*.

**Fig. 7:**
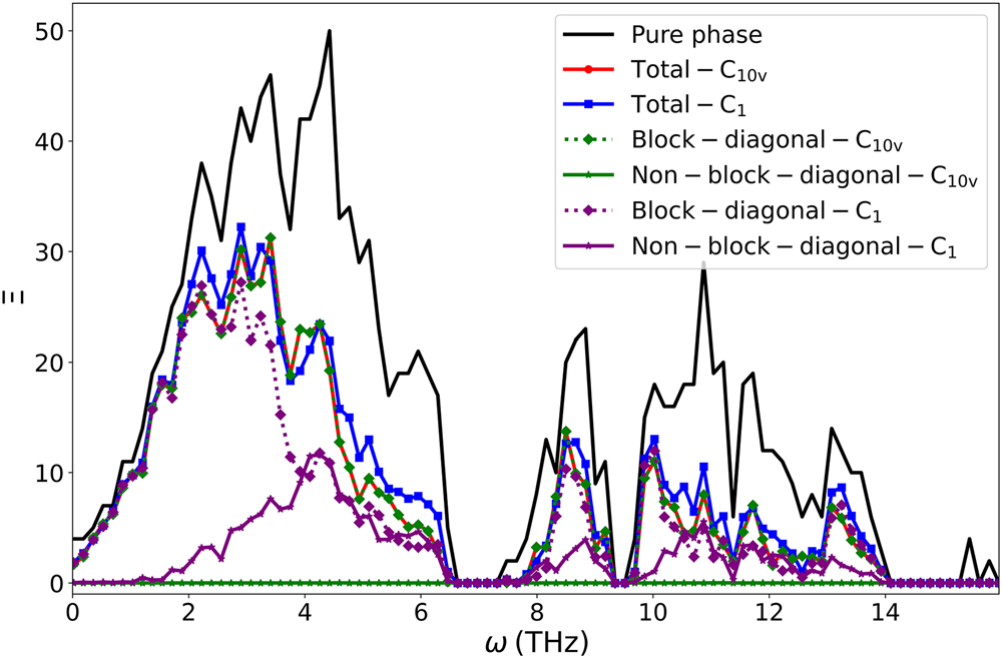
Contributions to the transmission from diagonal and off-diagonal blocks in the transmission matrices for different pristine or defect-laden configurations. The total transmission across a pristine segment is given in black, the transmission across a symmetric defect system in red, and the transmission across an asymmetric segment in blue. The separate contributions for the diagonal and off-diagonal blocks are shown with dotted and solid lines, respectively, where green and purple represent the symmetric and asymmetric systems. The solid red and dotted green lines overlap exactly.

For structures with broken symmetry, energy can access more transmission channels instead of being confined to the same irrep. In this way, although the irrep-preserving transmission probability decreases, the overall transmission increases due to compensation from the off-diagonal terms. As shown in Fig. 7, the transmission for the structure maintaining *C*_10*v*_ symmetry shows no contribution from the off-diagonal terms. In contrast, for the broken-symmetry structure *C*_1_, the off-diagonal terms contribute significantly and are even comparable to the diagonal terms in some frequency ranges. We can see that, in those areas with off-diagonal compensation, the total transmission coefficient of the *C*_1_ structure is higher than that of the *C*_10*v*_ structure.

**Fig. 8:**
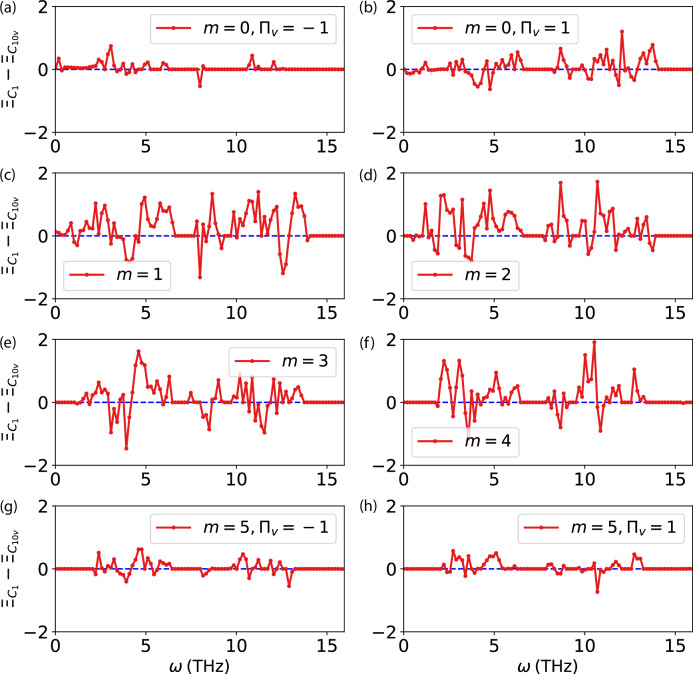
The transmittance differences between the systems with symmetry-breaking and symmetry-conserving defects for different irreps. The transmittance differences ${\Xi }_{{C}_{1}}-{\Xi }_{{C}_{10v}}$ are shown for the different parities for *m* = 0 in (**a**) and (**b**), for *m* ranging from 1 to 4 in (**c**–**f**), and for the different parities for *m* = 5 in (**g**) and (**h**).

In Fig. 8 we compare the transmissions of the *C*_1_ and *C*_10*v*_ structures by plotting their differences irrep by irrep. In the low-frequency range, transmission is predominantly influenced by modes with low *m* numbers, whereas larger values of *m* correspond to modes in higher frequency ranges. The difference between ${\Xi }_{{C}_{1}}$ and ${\Xi }_{{C}_{10v}}$ confirms that, across most frequency ranges, the more disordered structure exhibits a higher transmission probability.

Eight more comparisons between lower- and higher-symmetry configurations of this system are provided as part of the supplementary information, showing that these results are not fortuitous, and supporting our analysis in terms of the off-diagonal blocks of the transmission matrix.

## Discussion

The symmetry-resolved AGF method we have developed enables detailed computational analysis of how symmetry variations affect phonon transmission. By applying the symmetry projector technique, phonon branches are decomposed into distinct irreps, splitting the problem into many simpler ones and avoiding numerical artifacts. This decomposition yields real phonon modes categorized by irreps that can be directly used to compute the symmetry-adapted transmission.

The results for pristine nanotubes show that the additional quantum numbers assigned to vibrational modes when viewed through the lens of the line group of the system are not merely a mathematical tool to reduce the size of the matrix blocks involved in the workflow: they provide physical insight into the modes they label. Just as *q* indexes the linear quasimomentum (conjugate to the Cartesian coordinate along the longitudinal axis) by characterizing the transformation under a translation, *m* indexes the angular quasimomentum of the mode (conjugate to the angular coordinate around the same axis) by characterizing the transformation under a pure rotation. To further highlight this physical meaning, it is worth mentioning that for groups with a helical generator, there exists an alternative set of quantum numbers that eschew the usual linear quasimomentum in favor of bands indexed by a helical quasimomentum $\tilde{q}$ and a pure angular quasimomentum $\tilde{m}$ associated only with the operations of the point subgroup^24^. For commensurate systems like those treated here, there is a correspondence between the sets of irreps defined by (*q*, *m*) and those defined by $(\tilde{q},\tilde{m})$, but incommensurate helical systems only admit a description based on the latter. In those cases, instead of phonons propagating along the nanotube axis with a wave number taken from the usual Brillouin zone, the system’s vibrations are conceptualized as “helical phonons” traveling along helical arcs, and $\tilde{q}$ take values within a helical Brillouin zone. Note that in this work, we always opt for the (*q*, *m*) description because it integrates better with supercell-based calculations.

We also apply this method to a multi-layer WS_2_-MoS_2_ system where we modulate the structural symmetry by altering the arrangement of substitutional defects in the scattering region. Our findings show that high-symmetry defects constrain phonon scattering to occur within the same irreps, consistent with symmetry-imposed selection rules. In contrast, as the defect-induced symmetry is significantly reduced, additional phonon transmission channels, previously forbidden by selection rules, become possible, leading to an increase in both total transmission and thermal conductance.

The calculations presented in the previous section represent a purely harmonic level of description, and therefore fail to account for either the progressively more important effect of anharmonicity at higher temperatures or possible temperature-induced structural distortions causing deviations from symmetry. Our MD simulations allow us to capture anharmonicity to all orders and assess the robustness of our observations.

A first class of problems could emerge at finite temperatures due to the breakdown of structural symmetries. However, our MD results reveal that the time-averaged atomic positions at temperatures of 300K and 500K maintain the original symmetry to a tolerance of 0.01Å. At higher temperatures, the double-walled WS_2_-MoS_2_ system will occasionally exhibit a relative rotation between the weakly interacting inner and outer tubes, but this only causes a transition between two equivalent configurational minima with the same symmetry.

We also investigate the full thermal conductivity using Green-Kubo simulations. They reveal thermal conductivity values of (9.8  ± 0.8) W m^−1 ^K^-1^ and (6.1 ± 0.6) W m^−1 ^K^−1^ for the less and more symmetric defect-laden versions of the double-walled nanotube. This result confirms our findings from the transmission calculations and shows an even more significant difference in the total thermal conductivity when including higher-order scattering processes. While these MD simulations do not afford a similarly detailed look into the origin of those larger differences, their results do show that asymmetric defects can lead to an enhanced conductivity at finite temperatures compared to symmetric defects.

## Methods

### Construction of MoS_2_-WS_2_ nanotube structures

The starting configuration of MoS_2_-type nanotubes can be obtained by rolling up a strip of the corresponding 2D hexagonal lattice. That strip is defined^22^ by two chiral indices (*n*_1_, *n*_2_) that also determine the line group of the structure, and we carefully constrain the resulting coordinates so that it indeed belongs to the desired line group^24,33^. To further improve the quality of the result, we require that the distances between each chalcogenide atom and its three nearest-neighbor Mo atoms be equal to the bond length in the 2D monolayer structure. Since we apply this constraint directly to the symcell (the smallest unit that can generate the unit cell, and therefore the whole structure, through symmetry operations), it does not change the line group of the final structure. The starting configurations of multi-layer MoS_2_-WS_2_ nanotubes are built by combining single-wall nanotubes of MoS_2_ and WS_2_ with different diameters. Specifically, since the W-S and Mo-S bond lengths in the 2D monolayer structures are very similar (approximately^34,35^ 2.43 Å), we use the MoS_2_ lattice vectors to build the initial structure of double-walled nanotubes, after which we relax the atomic positions using the software and parameters discussed in the next subsection. The code for constructing nanotubes is available as part of our line-group-symmetry-analysis software, Pulgon-tools^36^. The effect of the relaxation is shown in the supplementary information.

### Density functional theory (DFT) calculations

We use the projector-augmented-wave formalism^37^ as implemented in the 6.4.0 release of VASP^38–40^, together with the Perdew-Burke-Ernzerhof approximation to the exchange and correlation energy^41^ and the DFT-D3 method with a Becke-Johnson damping function^42^ to approximately account for van-der Waals interactions. We choose 4*p*^6^5*s*^1^4*d*^5^, 6*s*^2^5*d*^4^, and 3*s*^2^3*p*^4^ as valence configurations for Mo, W, and S, respectively, a plane-wave cutoff energy of 500 eV, and 1 × 1 × 8, 1 × 1 × 2, and 2 × 2 × 1 Monkhorst-Pack k-point grids for single-walled nanotubes, double-walled nanotubes, and 2D structures, respectively. We moreover set an electronic convergence criterion of 10^−8^ eV for all VASP runs. For structural relaxations, we use a threshold of 10^−2 ^eV Å^−1^ for the forces.

### Machine-learning interatomic potential

Our MLIP is based on Allegro^31^. We use a force field with two hidden layers and take a cutoff radius *r*_cut_ = 7 Å. For the training, we choose an Adam^43^ optimizer with a learning rate of 5 × 10^−4^. which we run for 2500 epochs. The dimensions of the two-body latent multi-layer perceptron (MLP) are set to $\left(128,256,512\right)$. The latent and output MLPs contain a single layer each, with widths of 512 and 32, respectively. All three perceptrons use the Swish-1 activation function^44^.

**Fig. 9:**
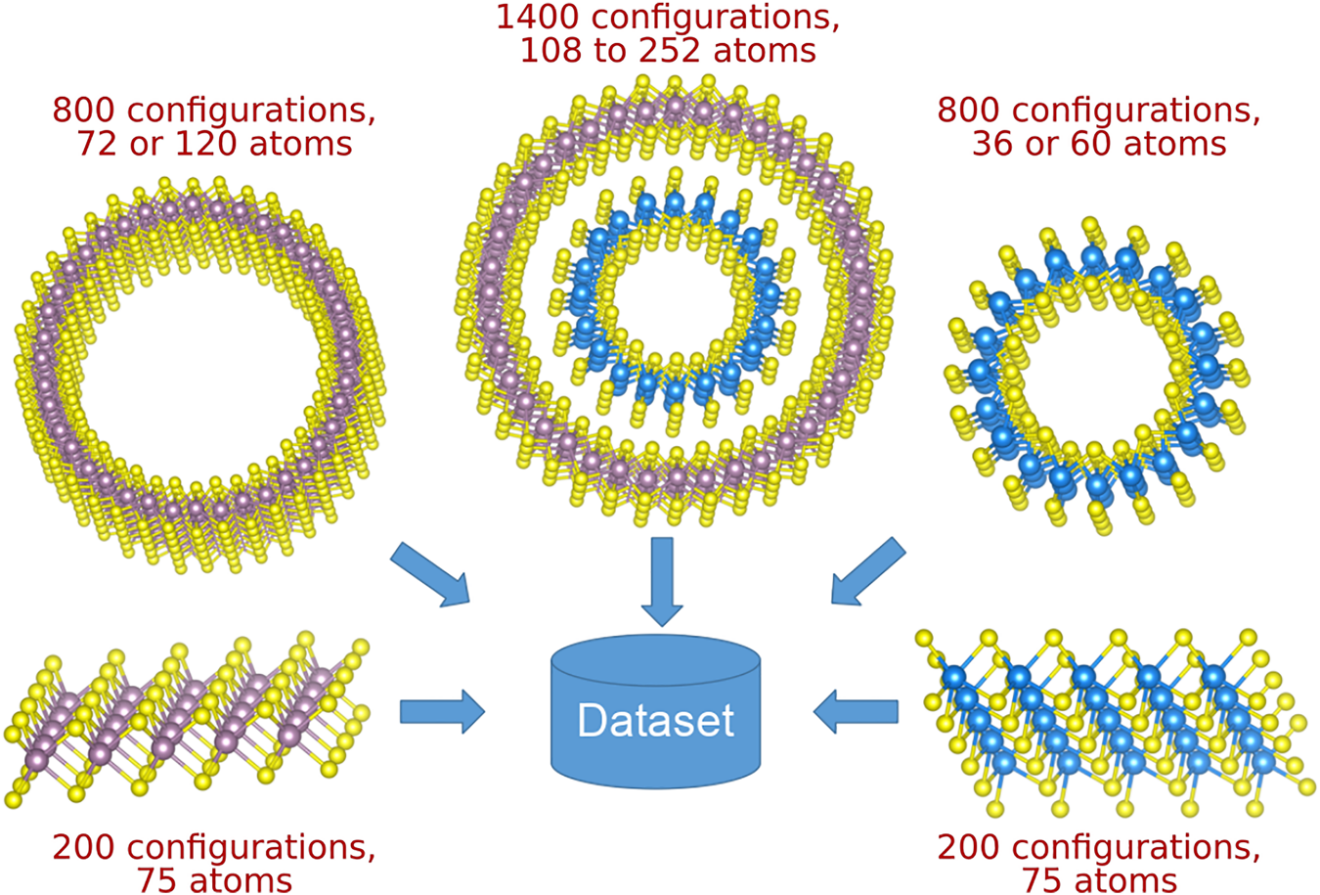
Outline of the structures used to build the ab initio dataset. The number of configurations and the respective ranges of system sizes are given in red text. The atomic visualizations were created with the VESTA software package^63^.

The first-principles dataset used for the development of our MLIP contains configurations of the 2D monolayers as well as single- and double-wall nanotubes relaxed via DFT. Each structure is generated by starting from the ground-state configuration and applying random atomic displacements following Gaussian distributions with standard deviations of 3 × 10^−2^ Å to 5 × 10^−2^ Å. Those displaced structures are then used in single-point DFT calculations to obtain their energies and the forces on each atom, which are added to the dataset. From a total of 3400 configurations, we allocate 3000 to the training set and 400 to the validation set. The detailed breakdown is given in Fig. 9.

### Interatomic force constants

To obtain the second-order IFCs that determine the vibrational modes of our structures, we first generate the original single- or double-walled nanotubes and relax them using the trained MLIP. Secondly, we create a minimal set of displaced supercell structures using Phonopy^45,46^. Finally, we calculate the forces in those configurations with the trained MLIP and reconstruct the IFC tensor with Phonopy.

The second-order approximation to the potential energy landscape of a system defined by its harmonic IFCs cannot violate the conservation of linear and angular momentum. This imposes several sets of linear constraints on the IFCs, known as the acoustic sum rules (ASRs). For fully periodic 3D systems, generally only the translational sum rules need to be considered: their violation leads to unphysical non-zero frequencies for the acoustic phonon branches at the *Γ* point. However, for subperiodic systems like multilayers and nanotubes, the Born-Huang rotational sum rules^47^ are also critical^23,48^. Specifically, in quasi-1D systems, they ensure the presence of four acoustic branches (instead of three) and the quadratic character of two of those near *Γ*. There are several published strategies to enforce the ASRs. Some take the form of post-processing steps, including a modified harmonic expansion in terms of explicitly scalar coordinates^23^, a projection of the “raw” harmonic IFCs onto an ASR-compliant reduced space (as implemented in Quantum ESPRESSO^48^), and the addition of a correction term to the raw IFCs, minimized subject to the symmetry constraints (a strategy used by hiPhive^49^). However, it is also possible to directly include the constraints as part of the set of linear equations that is solved to obtain the IFCs of all orders, as done by ALAMODE^50^. We implement our own version of the post-processing approach, focusing on performance for large sets of second-order IFCs with many constraints, and on compatibility with the additional constraint of short range for the IFCs that the AGF formalism requires.

Specifically, we apply a post-processing step to the raw force constants to enforce the full set of constraints while deviating as little as possible from the original result. We do so by solving a linearly constrained quadratic optimization problem:

1 $$\begin{array}{ll}{{\boldsymbol{H}}}_{{\rm{corrected}}}=\arg \mathop{\max }\limits_{{{\boldsymbol{H}}}_{{\rm{corrected}}}}\left\{\parallel {{\boldsymbol{H}}}_{{\rm{corrected}}}-{{\boldsymbol{H}}}_{{\rm{raw}}}{\parallel }^{2}\right\}\\ {\boldsymbol{C}}\cdot {{\boldsymbol{H}}}_{{\rm{corrected}}}=0\end{array}$$

Here, ***H*** is the matrix of harmonic IFCs, with elements ${H}_{ij}^{\alpha \beta }$ (Latin subscripts denoting atom numbers and Greek superscripts representing Cartesian directions), and ***C*** is the matrix containing the coefficients of all the linear constraints. The list of constraints we impose comprises the basic matrix transposition symmetry (${\Phi }_{ij}^{\alpha \beta }={\Phi }_{ji}^{\beta \alpha }$), the usual acoustic translational sum rules, and the Born-Huang rotational rules^47^. On top of these physically motivated constraints, we add a second set of linear equations to ensure that the IFCs are short-sighted and thus conform to the assumptions of the AGF workflow that we implement^20^. In particular, this requires that the system be divided into translationally equivalent blocks along its periodic direction and that the blocks are long enough that only first-neighbor-block interactions need to be taken into account. We thus require that ${\Phi }_{ij}^{\alpha \beta }=0$ if atoms *i* and *j* are in different, non-adjacent blocks and that the IFC submatrix connecting blocks *b* and *b* + 1 is equal to that connecting blocks *b* − 1 and *b*.

We solve the problem expressed by Eq. (1) using the highly efficient convex-optimization package CVXPY^51^, which calls the OSQP^52^ operator-splitting solver. We use the resulting high-quality IFCs in all our calculations.

### Mode-resolved AGF calculations

**Fig. 10:**
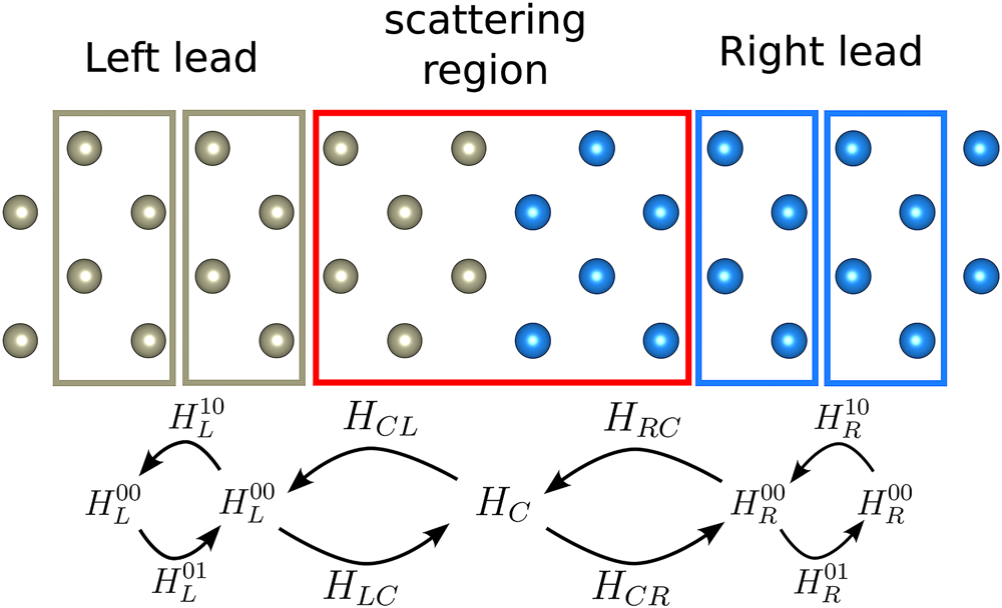
Schematic diagram of the structural partitioning used in the AGF method. The structural configurations of the left lead, scattering region, and right lead are shown here, along with the symbols for the IFC matrices whose shape they determine.

In the traditional AGF method^18,53^, the quasi-1D system is decomposed into three different parts: the left/right leads—two semi-infinite regions with discrete translation symmetry—and a scattering region where the interface is. This is schematically shown in Fig. 10. Accordingly, the IFC matrix *H* can be divided into a finite set of blocks, ${{\boldsymbol{H}}}_{L}^{00}$, ***H***_*C*_, ${{\boldsymbol{H}}}_{R}^{00}$, ${{\boldsymbol{H}}}_{{\rm{L}}}^{01}$, ${{\boldsymbol{H}}}_{{\rm{L}}}^{10}$, ***H***_LC_, ***H***_CL_, ***H***_CR_, ***H***_RC_, ${{\boldsymbol{H}}}_{{\rm{R}}}^{01}$, and ${{\boldsymbol{H}}}_{{\rm{R}}}^{10}$, which describe the interactions within a region or between different regions. The retarded surface Green’s functions of the uncoupled leads are calculated using decimation, an efficient real-space iterative process^53,54^. Those are:

2a $${{\boldsymbol{g}}}_{\alpha ,-}^{{\rm{ret}}}={[({\omega }^{2}+i\eta ){\boldsymbol{1}}-{{\boldsymbol{H}}}_{\alpha }^{00}-{{\boldsymbol{H}}}_{\alpha }^{10}{{\boldsymbol{g}}}_{\alpha ,-}^{{\rm{ret}}}{{\boldsymbol{H}}}_{\alpha }^{01}]}^{-1}$$

2b $${{\boldsymbol{g}}}_{\alpha ,+}^{{\rm{ret}}}={[({\omega }^{2}+i\eta ){\boldsymbol{1}}-{{\boldsymbol{H}}}_{\alpha }^{00}-{{\boldsymbol{H}}}_{\alpha }^{01}{{\boldsymbol{g}}}_{\alpha ,+}^{{\rm{ret}}}{{\boldsymbol{H}}}_{\alpha }^{10}]}^{-1}$$

and their Hermitian conjugates are ${{\boldsymbol{g}}}_{\alpha ,-}^{{\rm{adv}}}$ and ${{\boldsymbol{g}}}_{\alpha ,+}^{{\rm{adv}}}$, respectively. Here, *ω* is the vibration frequency of phonons, *α* is either *L* or *R* and refers to the left or right lead, and ret/adv indicates whether this is the retarded or advanced Green’s function, and  +/− describes whether the semi-infinite lead extends towards the right or the left, respectively. *η* is a small positive number enforcing the choice between ret and adv, and ***1*** is an identity matrix of the appropriate size.

The Green’s function in the central region is defined as:

3 $${{\boldsymbol{G}}}_{{\rm{C}}}^{{\rm{ret}}}={({\omega }^{2}{\boldsymbol{1}}-{{\boldsymbol{H}}}_{{\rm{C}}}-{\Sigma }_{{\rm{L}}}-{\Sigma }_{{\rm{R}}})}^{-1},$$

which involves the two self-energies ${\Sigma }_{L}={{\boldsymbol{H}}}_{{\rm{CL}}}{{\boldsymbol{g}}}_{L,-}^{{\rm{ret}}}{{\boldsymbol{H}}}_{{\rm{LC}}}$ and ${\Sigma }_{R}={{\boldsymbol{H}}}_{{\rm{CR}}}{{\boldsymbol{g}}}_{R,+}^{{\rm{ret}}}{{\boldsymbol{H}}}_{{\rm{RC}}}$.

The total transmission coefficient of the system can be calculated through the Caroli formula:

4 $$\Xi (\omega )={\rm{Tr}}[{{\mathbf{\Gamma }}}_{{\rm{R}}}{{\boldsymbol{G}}}_{{\rm{C}}}^{{\rm{ret}}}{{\mathbf{\Gamma }}}_{{\rm{L}}}{({{\boldsymbol{G}}}_{{\rm{C}}}^{{\rm{ret}}})}^{\dagger }]$$

where $${{\mathbf{\Gamma }}}_{{\rm{L}}}=i{{\boldsymbol{H}}}_{{\rm{L}}}^{10}({{\boldsymbol{g}}}_{{\rm{L}},-}^{{\rm{ret}}}-{{\boldsymbol{g}}}_{{\rm{L}},-}^{{\rm{adv}}}){{\boldsymbol{H}}}_{{\rm{L}}}^{01}$$ and $${{\mathbf{\Gamma }}}_{{\rm{R}}}=i{{\boldsymbol{H}}}_{{\rm{R}}}^{01}({{\boldsymbol{g}}}_{{\rm{R}},+}^{{\rm{ret}}}-{{\boldsymbol{g}}}_{{\rm{R}},+}^{{\rm{adv}}}){{\boldsymbol{H}}}_{{\rm{R}}}^{10}$$.

The mode-resolved AGF^19,20,55^ method improves upon the traditional AGF approach in that it uses the Bloch matrices to model the transmission of individual phonon modes. This enables a detailed analysis of the contributions of specific phonon modes to interfacial thermal transport.

The relevant Bloch matrices are constructed as follows:

5 $${{\boldsymbol{F}}}_{\alpha }^{{\rm{adv}}/{\rm{ret}}}{(-)}^{-1}={{\boldsymbol{g}}}_{\alpha ,-}^{{\rm{adv}}/{\rm{ret}}}{{\boldsymbol{H}}}_{\alpha }^{01}$$

The results are extracted from the solutions of the eigenvalue equations for the Bloch matrices,

6 $${{\boldsymbol{F}}}_{\alpha }^{{\rm{adv}}/{\rm{ret}}}{\left(-\right)}^{-1}{{\boldsymbol{U}}}_{\alpha }^{{\rm{adv}}/{\rm{ret}}}(-)={{\mathbf{\Lambda }}}_{\alpha }^{{\rm{adv}}/{\rm{ret}}}{\left(-\right)}^{-1}{{\boldsymbol{U}}}_{\alpha }^{{\rm{adv}}/{\rm{ret}}}(-),$$

where ${{\boldsymbol{U}}}_{\alpha }^{{\rm{adv}}/{\rm{ret}}}=({{\boldsymbol{e}}}_{1},{{\boldsymbol{e}}}_{2},...,{{\boldsymbol{e}}}_{n})$ are the matrices of normalized eigenvectors of ${{\boldsymbol{F}}}_{\alpha }^{{\rm{adv}}/{\rm{ret}}}$ and ${{\mathbf{\Lambda }}}_{\alpha }^{{\rm{adv}}/{\rm{ret}}}$ represent the corresponding diagonal matrices with their eigenvalues.

The number of rightward-going phonon channels in a perfect lead at frequency *ω* is given by:

7 $${N}_{\alpha }={\rm{Tr}}[{{\boldsymbol{V}}}_{\alpha }^{{\rm{adv}}}(-){\widetilde{{\boldsymbol{V}}}}_{\alpha }^{{\rm{adv}}}(-)]$$

where ${{\boldsymbol{V}}}_{\alpha }^{{\rm{adv}}/{\rm{ret}}}$ is the diagonal matrix that contains the group velocities of the eigenvectors in ${{\boldsymbol{U}}}_{\alpha }^{{\rm{adv}}/{\rm{ret}}}$ as its elements. ${\widetilde{{\boldsymbol{V}}}}_{\alpha }^{{\rm{adv}}/{\rm{ret}}}$ is also a diagonal matrix, whose elements are either the inverses of the corresponding diagonal elements of ${{\boldsymbol{V}}}_{\alpha }^{{\rm{adv}}/{\rm{ret}}}$ or zero (when those diagonal elements are also zero).

The mode-resolved transmission matrix from left to right is:

8 $$\begin{array}{rcl}{{\boldsymbol{t}}}_{{\rm{RL}}}&=&\frac{2i\omega }{\sqrt{{a}_{{\rm{R}}}{a}_{{\rm{L}}}}}{[{{\boldsymbol{V}}}_{{\rm{R}}}^{{\rm{ret}}}(+)]}^{1/2}{[{{\boldsymbol{U}}}_{{\rm{R}}}^{{\rm{ret}}}(+)]}^{-1}{{\boldsymbol{G}}}_{{\rm{RL}}}^{{\rm{ret}}}\\ &&\times {[{{\boldsymbol{U}}}_{{\rm{L}}}^{{\rm{adv}}}{(-)}^{\dagger }]}^{-1}{[{{\boldsymbol{V}}}_{{\rm{L}}}^{{\rm{adv}}}(-)]}^{1/2},\end{array}$$

involving the coupled retarded Green’s function ${{\boldsymbol{G}}}_{{\rm{RL}}}^{{\rm{ret}}}={[({\omega }^{2}+i\eta ){\boldsymbol{1}}-{{\boldsymbol{H}}}_{{\rm{L}}}]}^{-1}{{\boldsymbol{H}}}_{{\rm{RC}}}{{\boldsymbol{G}}}_{{\rm{C}}}^{{\rm{ret}}}{{\boldsymbol{H}}}_{{\rm{CL}}}{[({\omega }^{2}+i\eta ){\boldsymbol{1}}-{{\boldsymbol{H}}}_{{\rm{R}}}]}^{-1}$.

The transmission coefficient for each phonon channel *n* is written as:

9 $${\Xi }_{{\rm{L,n}}}(\omega )={[{{\boldsymbol{t}}}_{{\rm{RL}}}^{\dagger }{{\boldsymbol{t}}}_{{\rm{RL}}}]}_{nn}$$

### Symmetry-group-projector method

For each of the leads, the matrix of harmonic IFCs is block-diagonal on the basis of linear combinations of the atomic displacements adapted to the irreps of the line group. Since we only work with commensurate structures, it is possible to choose a set of irreps with well-defined wave numbers (*q*) along the nanotube axis. Thus, it is enough to focus on block-diagonalizing the dynamical matrix ***D***(*q*) at each value of *q*. To obtain the required basis, we first employ our line-group-symmetry-analysis software, Pulgon-tools^36^, to identify the line group L = Z ⋅ P and determine the character *χ*^*μ*^(*g*) for each group element *g* in each irrep *μ*. Here, Z denotes the generalized translation group, which may take the form of a helical group (C_Q_∣*f*) or a glide plane group (*σ*_v_∣*a*/2). The axial point group P belongs to one of the following seven types: C_*n*_, S_2*n*_, C_*nh*_, D_*n*_, C_*nv*_, D_*nd,*_ and D_*nh*_.

Next, we build the incomplete projection operator:

10 $${{\boldsymbol{P}}}^{\mu }=\frac{{d}_{\mu }}{| G| }\sum _{g}{\chi }^{\mu }{(g)}^{* }{\boldsymbol{S}}(g)$$

where *d*_*μ*_ is the degeneracy of the irrep *μ*, ∣*G*∣ is the order of the symmetry group *G*, and *χ*^(*μ*)^(*g*) is the character of the group element *g* in irrep *μ*; ***S***(*g*) is the matrix for operation *g*, defined by the transformation matrix of the atomic displacements in the simulation box (***M***) and two phase factors:

11 $${{\boldsymbol{S}}}_{ij}^{\alpha \beta }(g)={{\boldsymbol{M}}}_{ij}^{\alpha \beta }(g)\cdot {e}^{iq{T}_{z}}\cdot {e}^{iq\cdot ({r}_{i}^{z}-{r}_{j}^{z})}.$$

In turn, ***M*** is constructed by placing copies of the three-dimensional rotation matrix associated with *g* at the rows and columns defined by the atom permutation induced by said symmetry operation. The first factor, ${e}^{iq{T}_{z}}$, is associated with the translational component of the group operation *g*, and *T*_*z*_ is the *z* component of the translational vector (since we define the periodic direction to be *z*, the *x* and *y* components of the translation are always zero for line-group operations). For integer translational vectors this term is equal to 1 and can be ignored; however, in the case of helical symmetry operations in line groups, fractional translation vectors are possible, and the factor may become relevant. The second factor, ${e}^{iq}\cdot ({r}_{i}^{z}-{r}_{j}^{z})$, is related to the atomic positions of atom pairs *i* and *j* connected by the permutation part of *g*. Note that it is possible to include this phase in the expression of the dynamical matrix instead, in which case it does not need to be added here.

We then extract an orthonormal basis $({{\boldsymbol{b}}}_{1},{{\boldsymbol{b}}}_{2}\ldots {{\boldsymbol{b}}}_{{d}_{\mu }})$ for the column space of the projection operator ***P***^*μ*^. The symmetry-adapted basis that block-diagonalizes ***D***(*q*) is built by concatenating all those bases.

### Symmetry-adapted transmission

To develop a symmetry-adapted version of AGF we use the symmetry-adapted basis to separate the eigenmodes belonging to each irrep before proceeding to calculate the group-velocity matrices and the mode-resolved transmission ${{\boldsymbol{V}}}_{\alpha }^{{\rm{adv}}/{\rm{ret}}}$.

From the eigenvalues *Λ* we can obtain the wave number *q*_*n*_ for each mode:

12 $${q}_{n}=\frac{1}{{a}_{\alpha }}{\cos }^{-1}{\rm{Re}}{[{{\mathbf{\Lambda }}}_{\alpha }^{{\rm{adv}}/{\rm{ret}}}]}_{nn},$$

where *a*_*α*_ is the cell length of the *α* (L or R) lead. Degenerate modes are detected by grouping together numerically equal values of *q*_*n*_ within a tolerance. While eigenvectors for non-degenerate modes belong to an irrep and do not require post-processing, degenerate sets of modes can come out as linear combinations mixing different irreps. Therefore, it is necessary to disentangle them by calculating ${\rm{kernel}}([{{\boldsymbol{e}}}_{1},...,{{\boldsymbol{e}}}_{n},-{{\boldsymbol{b}}}_{1},...,-{{\boldsymbol{b}}}_{{d}_{\mu }}])$. The first *n* components of each element of an orthogonal basis of that kernel serve as the set of coefficients $\left\{{\lambda }_{i}^{\mu }\right\}$ for a linear combination of the $\left\{{{\boldsymbol{e}}}_{i}\right\}$. The new eigenvector ${{\boldsymbol{E}}}^{\mu }={\sum }_{i}{\lambda }_{i}^{\mu }{{\boldsymbol{e}}}_{{\boldsymbol{i}}}$ lies in the space spanned by the $\left\{{{\boldsymbol{b}}}_{j}\right\}$ which correspond to the irrep *μ*.

**Fig. 11:**
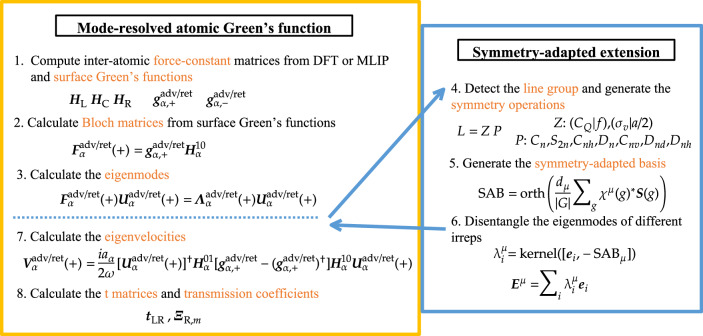
Flowchart illustrating the additional steps in the mode-resolved atomic Green’s function method. The left block illustrates the steps required for the general model-resolved AGF, while the right block outlines the extended procedure to detect the line group and obtain the eigenmodes corresponding to specific irreps.

A detailed scheme of the symmetry-adapted extension of the mode-resolved AGF technique is shown in Fig. 11.

### Thermal conductance

The thermal conductance for the material is calculated in the Landauer formalism as^14,56^:

13 $$\sigma (T)=\frac{1}{2\pi }\mathop{\int}\nolimits_{0}^{{\omega }_{\max }}\hslash \omega \frac{\partial n(\omega ,T)}{\partial T}\Xi (\omega )d\omega .$$

Here, *n*(*ω*, *T*) is the Bose-Einstein distribution and *Ξ*(*ω*) is the transmission coefficient.

### Finite-temperature MD simulations

Equilibrium MD simulations are employed to analyze the finite-temperature configurations and calculate the thermal conductivity of the systems. Supercells containing five layers of the nanotube unit cell are used, where one of the layers contains the symmetric or asymmetric defect configurations equivalent to those used for the transmission calculations. This leads to a lattice parameter in the periodic direction of 27.73Å and to a simulation cell with 900atoms. Specifically, to obtain the thermal conductivity, Green-Kubo simulations are employed at a temperature of 300K with a time step of 1fs. For each system, 15 independent simulations are performed with different velocity initialization seeds and an equilibration period of 20ps in an NVT ensemble guided by a Langevin thermostat. The heat flux is subsequently computed at every time step during a 200 ps period in NVE ensemble. The time integration is carried out by the atomic simulation environment (ASE)^57^, and the full heat flux is computed using

14 $${\bf{J}}=\frac{1}{V}\sum _{jk}{{\bf{r}}}_{kj}\left(\frac{\partial U}{\partial {{\bf{r}}}_{jk}}\cdot {{\bf{v}}}_{k}\right)+\frac{1}{V}\mathop{\sum}\limits_{j}{E}_{j}{{\bf{v}}}_{j},$$

where **r**_*kj*_ is the distance vector between atom *j* and *q*, **v**_*k*_ is the atomic velocity, *U* the potential energy of the system, *V* is the system volume, and *E*_*j*_ is the energy of atom *j*. The derivative in the first term is obtained via automatic differentiation over the Allegro model, which has been suggested as a computationally efficient method to obtain the heat flux for local MLIPs^58^.

The autocorrelation function of the computed heat flux in the periodic direction is then used to evaluate the thermal conductivity *κ* with its integral using the Green-Kubo formalism,

15 $${\mathbf{\kappa }}=\frac{V}{{k}_{B}{T}^{2}}\mathop{\int}\nolimits_{0}^{\infty } \langle{\bf{J}}(t){\bf{J}}(0) \rangle{\rm{d}}t.$$

However, Green-Kubo integrals can be very noisy and difficult to converge. Thus, we employ cepstral analysis for the evaluation as described in refs. ^59–61^. This approach is based on denoising the power spectrum of the heat flux to obtain a more accurate thermal conductivity value at lower simulation times. The full details of our approach and the tools required to implement it can be found in ref. ^61^. For this application, a cutoff of the power spectrum has to be chosen and is set to 0.5THz after careful consideration. The nanotube volume is computed based on the total volume occupied by the Van-der-Waals spheres around the individual atoms. For convergence tests, see Supplementary comment 4.

To investigate whether the defect-laden systems maintain their symmetry at elevated temperature, we employ MD simulations using settings analogous to those used for the Green-Kubo simulations and compute the average atomic positions during the NVE part of the simulation at 300 K and 500 K.

To assess the quality of the MLIP at finite temperatures, MD simulations are performed to generate MD trajectories for the defect-laden and defect-free nanotube systems in the NVT ensemble established by a Langevin thermostat for 10,000 1fs time steps at 300 K. We sample 50 configurations at even intervals between 5000 and 10,000 steps for each of the two structures, which are subsequently computed with DFT to allow the comparison.

## Supplementary information

Supplementary Information

## Data Availability

The DFT dataset, the Allegro MLIP model, and the double-walled nanotube structures are available on Zenodo (10.5281/zenodo.17279460).

## Code availability

The code for our symmetry-adapted AGF is available at https://github.com/pulgon-project/transmission. Related line group symmetry detection tools for quasi-1D systems are available at https://github.com/pulgon-project/pulgon_tools. The code to compute the heat flux for the Allegro model is available at https://github.com/pulgon-project/nequip-flux-calculator.
