# Supplementary material for: Ab-initio heat transport in defect-laden quasi-1D systems from a symmetry-adapted perspective
Source: NPJ Comput Mater. 2026 Jan 9;12(1):19. doi: 10.1038/s41524-025-01866-1 (PMC12789030; doi:10.1038/s41524-025-01866-1)
Supplement: Supplementary file 1 — Supplementary Information [file 41524_2025_1866_MOESM1_ESM.pdf]

Supplementary information for *Ab-initio heat transport in defect-laden quasi-1D systems from a symmetry-adapted perspective*

Yu-Jie Cen,[1] Sandro Wieser,[1] Georg K. H. Madsen,[1] and Jesús Carrete[2,*]
[1] *Institute of Materials Chemistry, TU Wien, A-1060 Vienna, Austria*
[2] *Instituto de Nanociencia y Materiales de Aragón, CSIC-Universidad de Zaragoza, E-50009 Zaragoza, Spain*
[*] jcarrete@gmail.com

October 24, 2025

Supplementary comment 1:

Additional comparisons between defect-laden systems with different symmetries

Here we show results for eight additional pairs of defect-laden configurations, in order to demonstrate that the results presented in the main text are not a coincidence. We consider two kinds of defects: (1) Mo-W substitutional defects and (2) S vacancies. In the case shown in Fig. 1, 10 W atoms are substituted for Mo atoms in the outer nanotube to build C_{10v} and C_1 structures. Since the number of defects is relatively small, the resulting decrease in transmission compared with the pristine nanotube is also minor, and the influence of symmetry on thermal conductance is not as apparent. In the case shown in Fig. 2, 10 Mo atoms are used to replace W atoms in the inner layer nanotube. It can be observed that the effect of the inner nanotube is relatively insignificant compared with that of the outer nanotube. In the cases shown in Fig. 3 and Fig. 4, there are 20 defect atoms and the transmission decreases more substantially. These two cases exhibit similar behavior to that discussed in the main text. In Fig. 5, the symmetry of the structure is partially broken, leading to slightly lower off-diagonal transmission blocks in the C_{5v} structure than in the C_1 structure. This indicates that the selection rules are partially relaxed in the C_{5v} configuration.

As for the cases involving S vacancies, some configurations lead to severe distortion or even structural collapse. We only show the examples that can be stably maintained under the MLIP optimization. In the case shown in Fig. 6, 10 S atoms connected to Mo atoms are removed from the outermost layer; the difference in thermal conductance between A and B is not significant. In the case shown in Fig. 7, when 10 S atoms connected to Mo atoms are removed from the inner layer, the thermal conductance does not change significantly either. When 10 S atoms are removed individually from the outermost and inner layers, as shown in Fig. 8, the gap in thermal conductance between the C_{10v} and C_1 configurations increases further.

Overall, in the (10,0)-(20,0) WS_2-MoS_2 double-layer nanotube, heat transfer is mainly influenced by the outer MoS_2 layer. Moreover, the effects of symmetry become significant only when defect density is high and transmission is reduced to a noticeable degree. However, in every case the higher-symmetry configuration has a lower conductance (even if only slightly so) and that discrepancy can be traced to the contribution of the off-diagonal blocks to the transmission, supporting the conclusions presented in the main text.

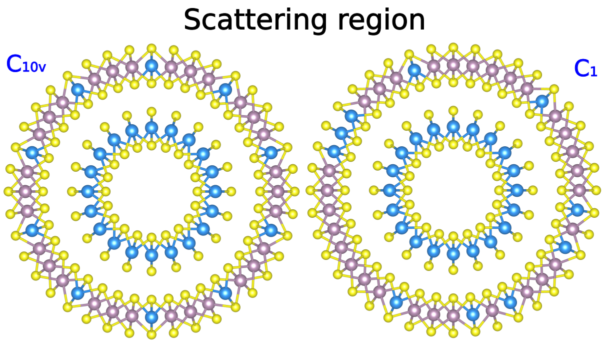

(a)

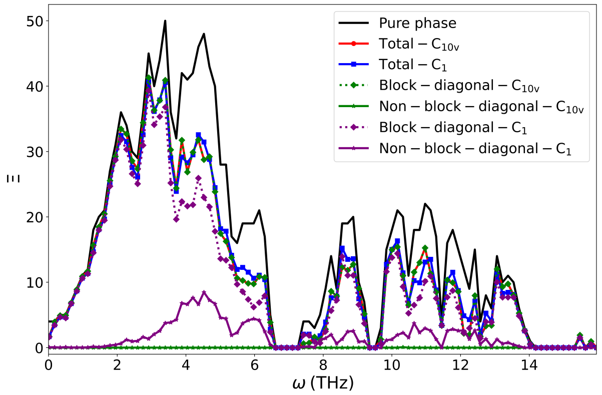

(b)

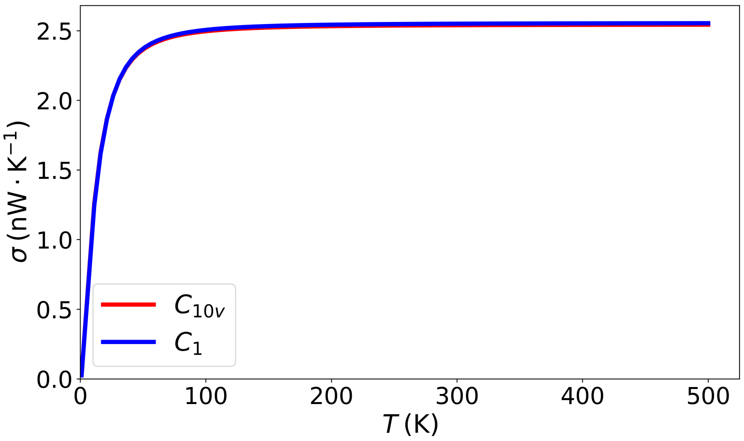

(c)

Supplementary figure 1: **Additional case 1: 10 Mo atoms are substituted by W atoms in the outer-layer nanotube to form the C_{10v} and C_1 configurations.** (a) Cross-sectional views of the two defect-laden configurations with different symmetries. (b) Contributions to the transmission from diagonal and off-diagonal blocks in the transmission matrix for different pristine or defect-laden configurations. (c) Temperature dependence of the thermal conductances for the two defect-laden structures. The atomic visualizations were created with the VESTA software package [1].

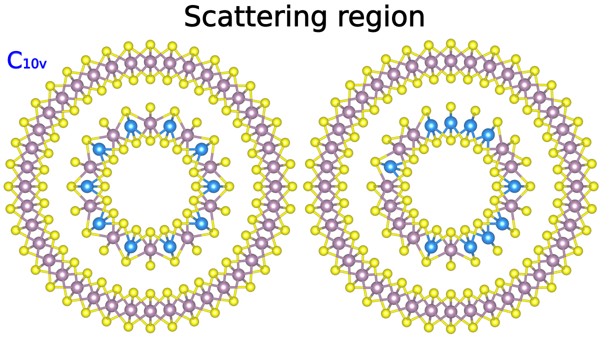

(a)

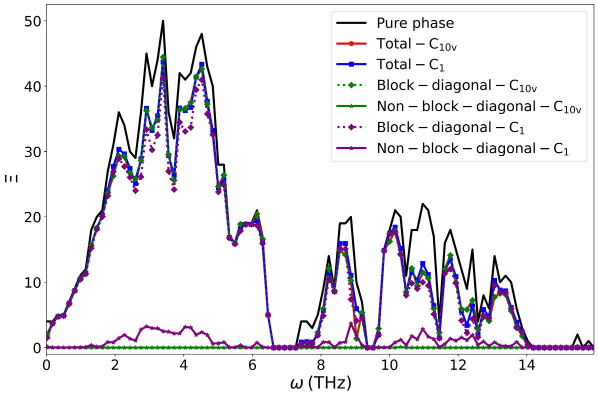

(b)

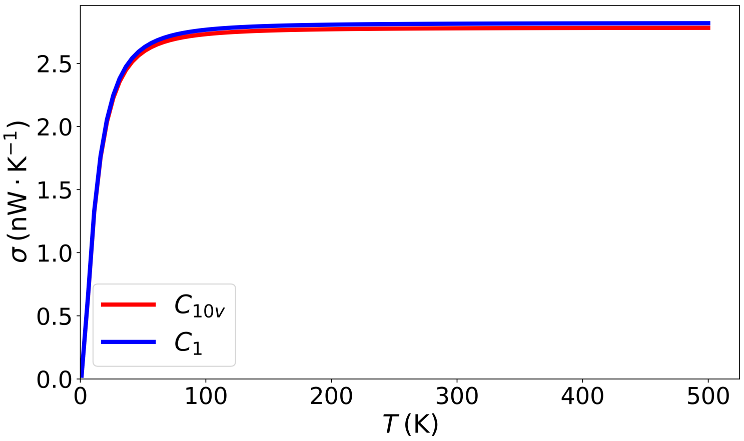

(c)

Supplementary figure 2: **Additional case 2: 10 W atoms are substituted by Mo atoms in the inner-layer nanotube to form the C_{10v} and C_1 configurations.** (a) Cross-sectional views of the two defect-laden configurations with different symmetries. (b) Contributions to the transmission from diagonal and off-diagonal blocks in the transmission matrix for different pristine or defect-laden configurations. (c) Temperature dependence of the thermal conductances for the two defect-laden structures. The atomic visualizations were created with the VESTA software package [1].

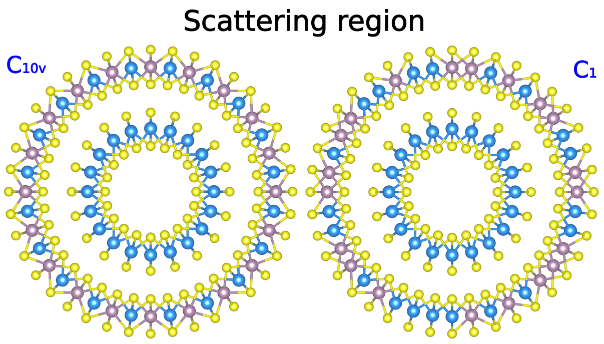

(a)

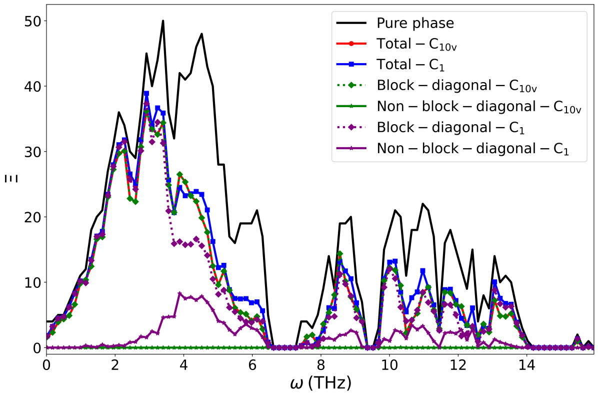

(b)

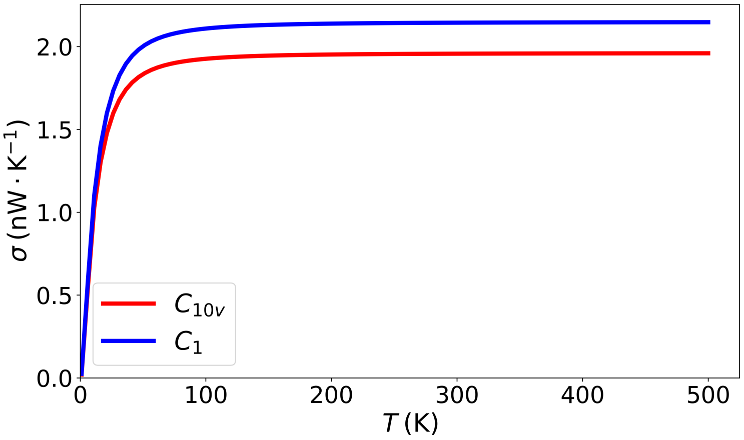

(c)

Supplementary figure 3: **Additional case 3: 20 Mo atoms are substituted by W atoms in the outer-layer nanotube to form the C_{10v} and C_1 configurations.** (a) Cross-sectional views of the two defect-laden configurations with different symmetries. (b) Contributions to the transmission from diagonal and off-diagonal blocks in the transmission matrix for different pristine or defect-laden configurations. (c) Temperature dependence of the thermal conductances for the two defect-laden structures. The atomic visualizations were created with the VESTA software package [1].

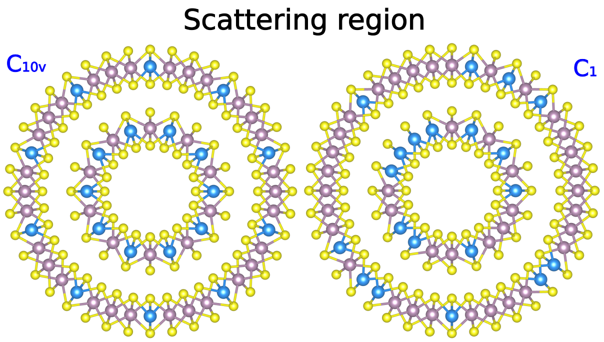

(a)

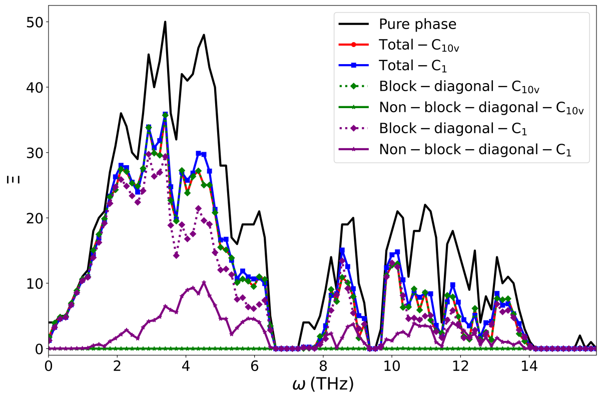

(b)

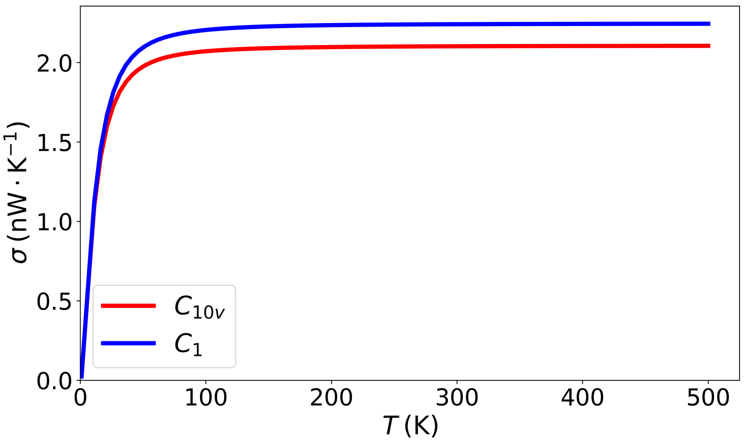

(c)

Supplementary figure 4: **Additional case 4: 10 Mo atoms are substituted by W atoms in the outer-layer nanotube and 10 W atoms are substituted by Mo atoms in the inner-layer nanotube to form the C_{10v} and C_1 configurations.** (a) Cross-sectional views of the two defect-laden configurations with different symmetries. (b) Contributions to the transmission from diagonal and off-diagonal blocks in the transmission matrix for different pristine or defect-laden configurations. (c) Temperature dependence of the thermal conductances for the two defect-laden structures. The atomic visualizations were created with the VESTA software package [1].

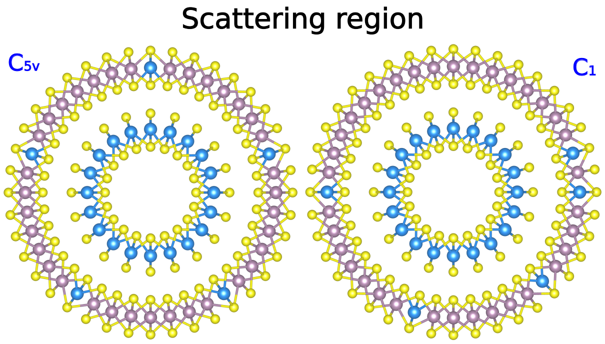

(a)

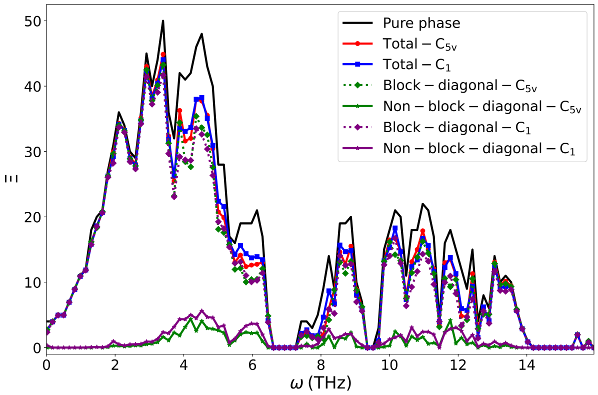

(b)

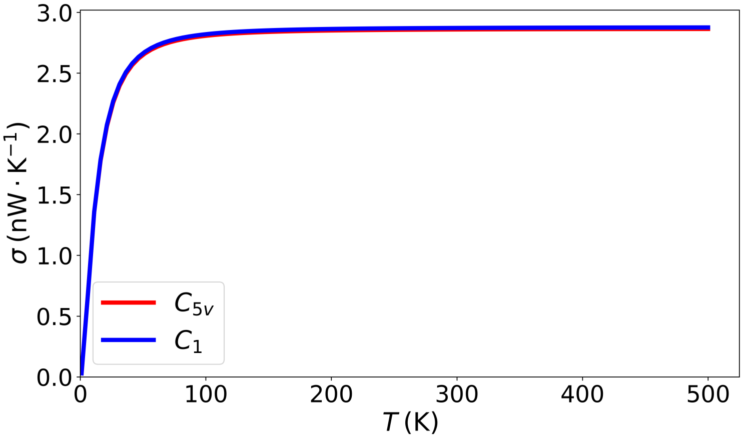

(c)

Supplementary figure 5: **Additional case 5: 5 Mo atoms are substituted by W atoms in the outer-layer nanotube to form the C_{5v} and C_1 configurations.** (a) Cross-sectional views of the two defect-laden configurations with different symmetries. (b) Contributions to the transmission from diagonal and off-diagonal blocks in the transmission matrix for different pristine or defect-laden configurations. (c) Temperature dependence of the thermal conductances for the two defect-laden structures. The atomic visualizations were created with the VESTA software package [1].

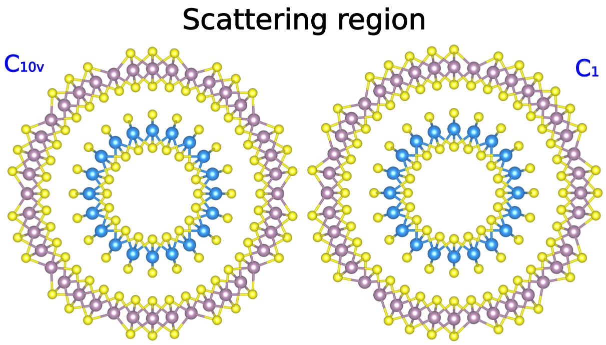

(a)

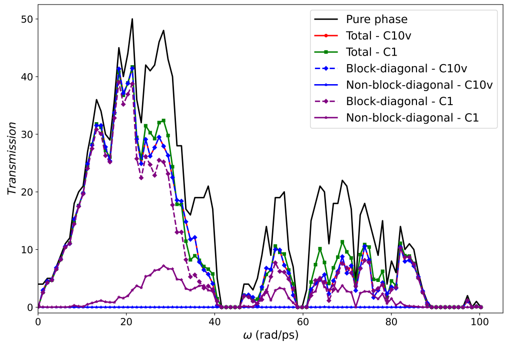

(b)

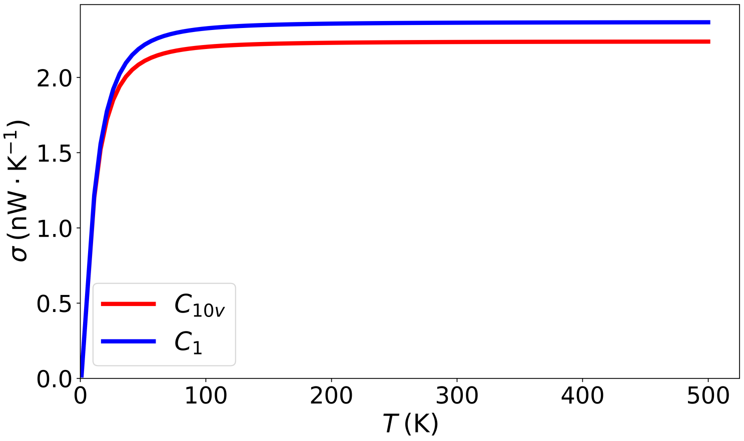

(c)

Supplementary figure 6: **Additional case 6: 10 S atoms are removed from the outermost layer connected to Mo atoms to form the C_{10v} and C_1 configurations.** (a) Cross-sectional views of the two defect-laden configurations with different symmetries. (b) Contributions to the transmission from diagonal and off-diagonal blocks in the transmission matrix for different pristine or defect-laden configurations. (c) Temperature dependence of the thermal conductances for the two defect-laden structures. The atomic visualizations were created with the VESTA software package [1].

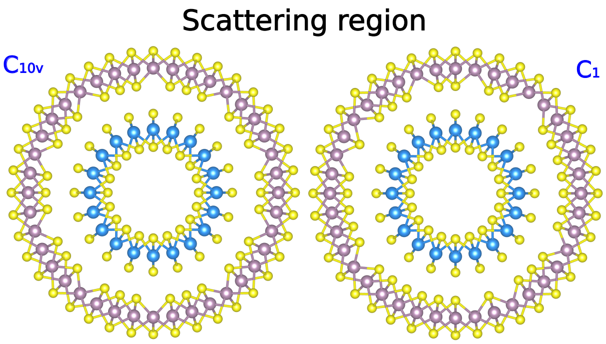

(a)

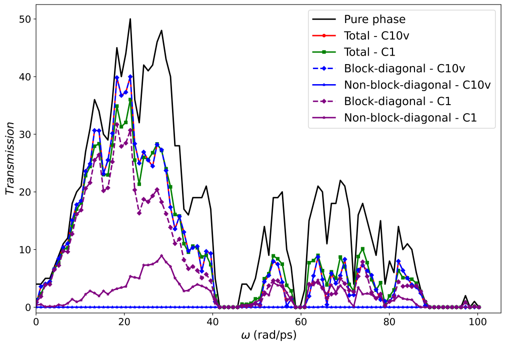

(b)

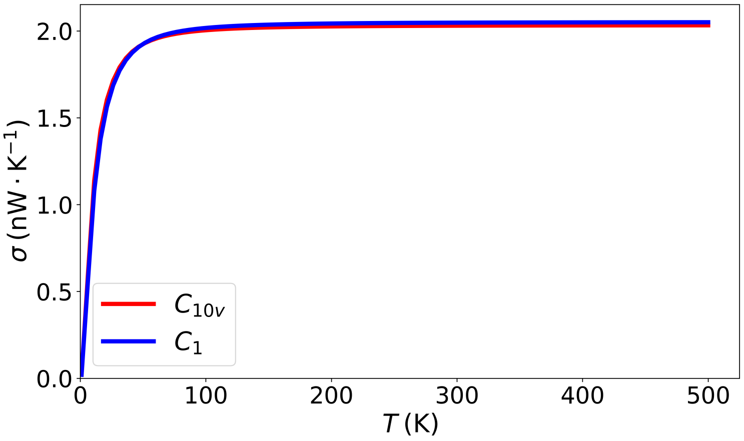

(c)

Supplementary figure 7: **Additional case 7: 10 S atoms are removed from the inner layer connected to Mo atoms to form the C_{10v} and C_1 configurations.** (a) Cross-sectional views of the two defect-laden configurations with different symmetries. (b) Contributions to the transmission from diagonal and off-diagonal blocks in the transmission matrix for different pristine or defect-laden configurations. (c) Temperature dependence of the thermal conductances for the two defect-laden structures. The atomic visualizations were created with the VESTA software package [1].

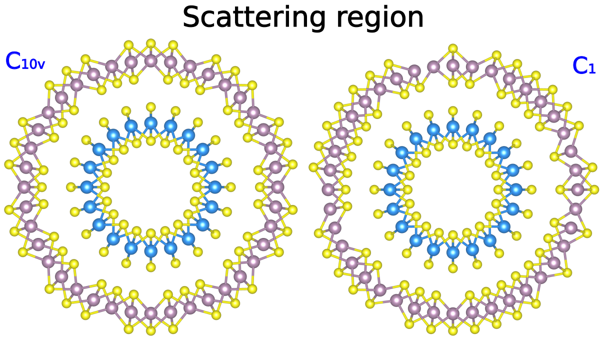

(a)

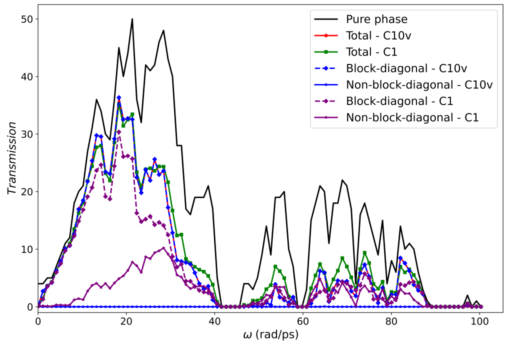

(b)

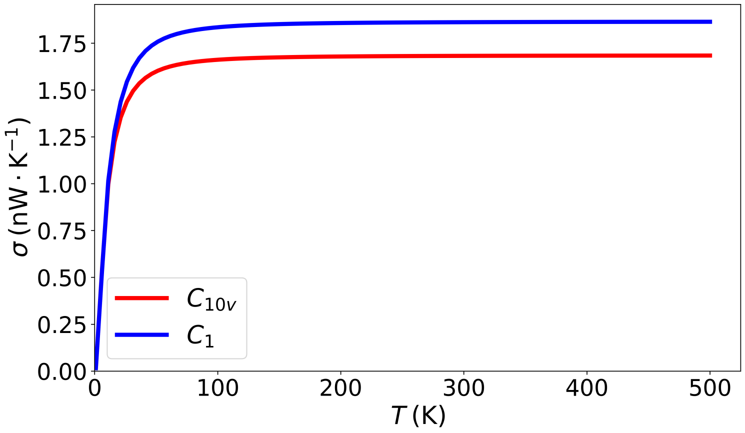

(c)

Supplementary figure 8: **Additional case 8: 10 S atoms are removed from the outermost and inner layer individually connected to Mo atoms to form the C_{10v} and C_1 configurations.** (a) Cross-sectional views of the two defect-laden configurations with different symmetries. (b) Contributions to the transmission from diagonal and off-diagonal blocks in the transmission matrix for different pristine or defect-laden configurations. (c) Temperature dependence of the thermal conductances for the two defect-laden structures. The atomic visualizations were created with the VESTA software package [1].

Supplementary comment 2:

Relaxation of the double-layer structure

All calculations for the double-walled nanotube were performed on a relaxed structure, obtained using the DFT code and parameters discussed in the main text. As shown in Fig. 9, a relative displacement between the inner and outer nanotube along the long axis of the structure as a result of energy minimization was detected.

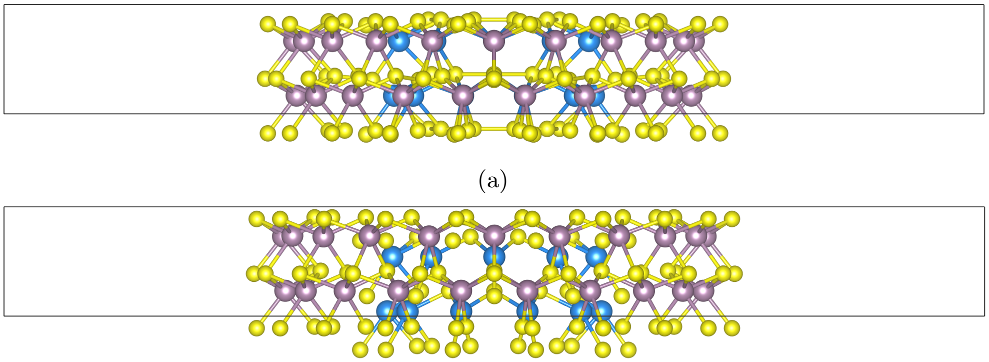

(a)

(b)

Supplementary figure 9: **Views of the double-walled nanotube before and after relaxing the atomic positions using density functional theory.** (a) Before relaxation and (b) after relaxation. The atomic visualizations were created with the VESTA software package [1].

Supplementary comment 3:

Performance of the Allegro MLIP on structures from finite-T trajectories

Here, we show the performance of the Allegro MLIP based on structures that were obtained from MD as described in the main text. The results for the pristine structure are shown in Fig. 10, with those for the defect-laden case displayed in Fig. 11.

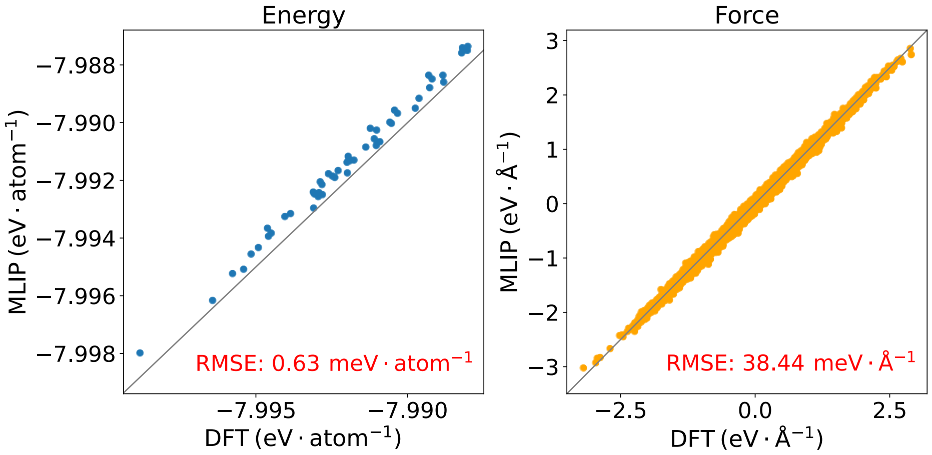

Supplementary figure 10: **Performance of the MLIP on MD trajectories of the pristine double-walled WS_2-MoS_2 nanotube.** We show the potential energy and forces for the structures sampled from the room-temperature (300 K) MD trajectory structures.

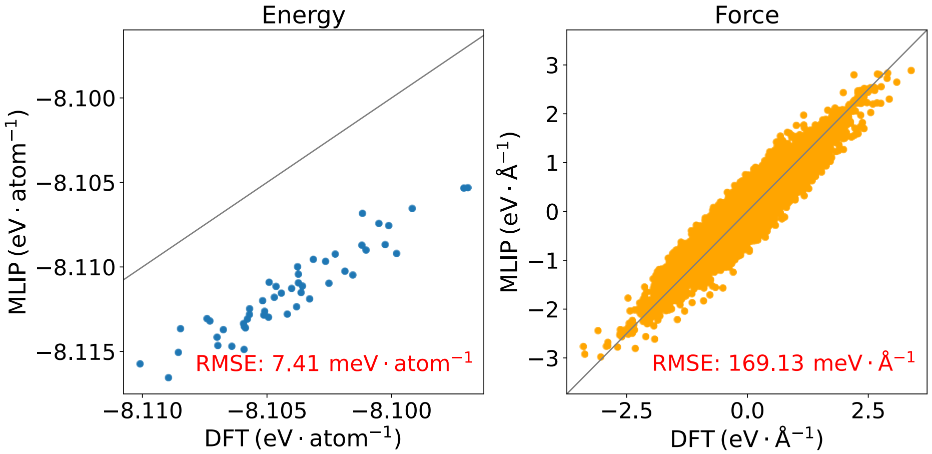

Supplementary figure 11: **Performance of the MLIP on molecular dynamics trajectories of the defect-laden double-walled WS_2-MoS_2 nanotube.** We show the potential energy and forces for the structures sampled from the room-temperature (300 K) MD trajectory structures.

Supplementary comment 4:

Green-Kubo simulations to obtain the thermal conductivity

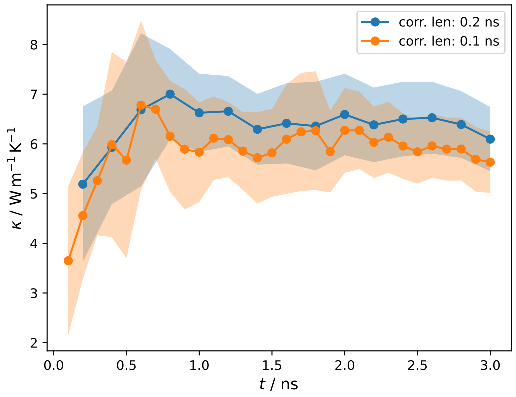

(a)

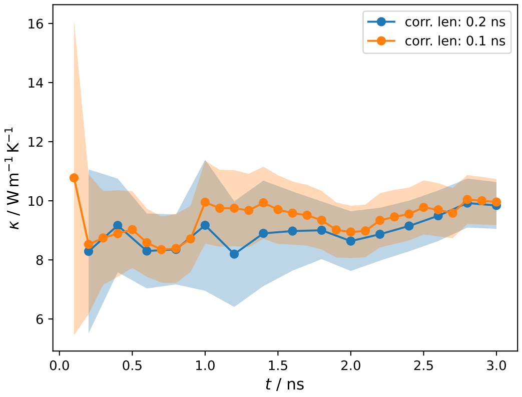

(b)

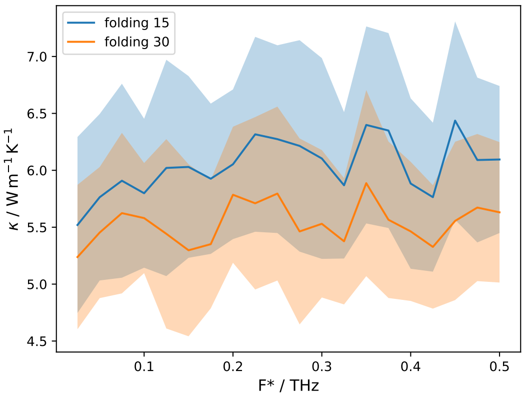

(c)

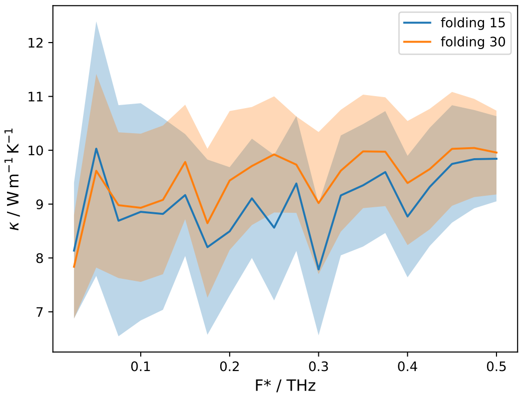

(d)

Supplementary figure 12: **Convergence plots for the thermal conductivity from Green-Kubo simulations.** Time convergence for the double-walled nanotube system containing the C_{10v}-symmetric defect (a) or the asymmetric defect (b). Differences based on the choice of cutoff for the double-walled nanotube system containing the C_{10v}-symmetric defect (c) and the asymmetric defect (d).

Figure 12 shows the convergence behavior of the Green-Kubo simulations. The autocorrelation functions were averaged over the 15 independent simulations (correlation time of 200 ps) or over twice that number after splitting each trajectory into two pieces (correlation time of 100 ps). For the final values in the main manuscript, a correlation time of 200 ps was chosen. The same correlation-time dependence is reflected in the cutoff analysis in Fig. 12c and d, where the number of folds indicates over how many pieces the averaging was performed. The results are given for the entire simulation time and it is clearly observed that the choice of cutoff is of limited significance within the predicted error. The cutoff was set to low values, as the objective is to obtain the denoised value at a frequency of 0 THz.

References

[1] Momma, K. & Izumi, F. Vesta3 for three-dimensional visualization of crystal, volumetric and morphology data. *J. Appl. Crystallogr.* **44**, 1272–1276 (2011).
